# Evaluating the Efficacy of Gum Arabic-Zinc Oxide Nanoparticles Composite Coating on Shelf-Life Extension of Mandarins (cv. Kinnow)

**DOI:** 10.3389/fpls.2022.953861

**Published:** 2022-07-22

**Authors:** Kwanele Andy Nxumalo, Olaniyi Amos Fawole, Oluwatobi Samuel Oluwafemi

**Affiliations:** ^1^Postharvest Research Laboratory, Department of Botany and Plant Biotechnology, University of Johannesburg, Johannesburg, South Africa; ^2^Department of Chemical Sciences (Formerly Applied Chemistry), University of Johannesburg, Johannesburg, South Africa; ^3^Centre for Nanomaterials Science Research, University of Johannesburg, Johannesburg, South Africa

**Keywords:** postharvest technology, green synthesis, South Africa, citrus, nanocomposites, edible coatings

## Abstract

Restricted postharvest application of synthetic fungicides in maintaining the quality of citrus fruits has led to a search for alternative postharvest treatments. This study evaluated the efficacy of gum arabic (GA) enriched with green synthesized zinc oxide nanoparticles (ZnO-NPs) in maintaining the postharvest quality of mandarin (cv. Kinnow). ZnO-NPs were synthesized using *Bidens pilosa* leaf extract and incorporated into GA (2% w/v) at 0, 0.25, 0.5, and 1% to form composite coatings: GA, GA + ZnO-NP 0.25%, GA + ZnO-NP 0.5% and GA + ZnO-NP 1%, respectively. Fruit were dipped for 3 min in the respective coatings, with untreated fruit used as control. Fruit were air-dried, packed in commercial cartons, and stored at 5 ± 1°C and 90 ± 5% relative humidity (RH) for 40 days and observed at 10 days intervals, plus 5 days at 20 ± 5°C and 65 ± 5% RH to determine the incidence of physiological disorders. GA + ZnO-NP showed promise as an alternative postharvest treatment for controlling postharvest physiological disorders associated with ‘Kinnow’ mandarin. For instance, GA + ZnO-NP 0.5% markedly minimized weight loss (9.2%), electrolyte leakage (43.8%) and chilling injury incidence (5.4%) compared to control (weight loss; 33.3%, electrolyte leakage; 90.3% and chilling injury incidence; 41.5%) at the end of the storage. GA + ZnO-NP 1% significantly alleviated rind pitting, with 13.2% incidence compared to 45.2% rind pitting incidence in the control fruit. This was due to significantly higher phytochemical and antioxidant capacity and reduced antioxidant enzyme degradation in coated fruit than in control. In conclusion, gum arabic coating enriched with ZnO-NPs at concentrations between 0.5 and 1% is recommended as a viable option to maintain the quality of ‘Kinnow’ mandarin fruit during cold storage.

## Introduction

Food and Agriculture Organization (FAO) reported that more than 50% of the fresh produce is destroyed yearly in developing countries because of postharvest losses (Food and Agriculture Organization of the United Nations [FAO], 2017). This results in reduced export value of the crops, foreign exchange, and gross domestic product (GDP) of those countries that rely on these exported commodities for trade. Citrus fruits are among the most sought-after fruits worldwide, mainly recognized for their high bioactive compounds such as phenolics, antioxidants, and other health-related properties (Lado et al., 2018; Department of Agriculture, Forestry and Fisheries [DAFF], 2021). However, fruit quality degradation during long-term cold storage affects the quality of these bioactive compounds, thus resulting in food loss and waste (Connor et al., 2002; Arendse et al., 2014). Like any citrus species, ‘Kinnow’ mandarin fruit has a relatively long postharvest life; however, they are prone to rind physiological disorders and decay because of fungal infections (Agustí et al., 2001; Lafuente and Sala, 2002; Zacarias et al., 2020). This reduces their market value, resulting in substantial economic losses. The control of these postharvest losses is difficult because it relies on the use of fungicides such as imazalil and thiabendazole, etc., which are a cause of concern to the public and the environment at large (Ncama et al., 2018; Riva et al., 2020). Commercial citrus waxes (usually based on oxidized polyethylene) are commonly used in the citrus industry to replace the natural wax removed from fruit surfaces during the washing and handling phase (Porat et al., 2005; Riva et al., 2020). This helps develop a barrier to water loss and gas (CO_2_ and O_2_) exchange, thus maintaining the quality of the fruit (Riva et al., 2020). These waxes are often incorporated with synthetic fungicides; however, because of their reported detrimental effects on human health, the environment and aquatic life, their use has been banned in many developed countries (Valencia-Chamorro et al., 2010; Nxumalo et al., 2021). As a result, the commercial success of the citrus industry is threatened. Therefore, there is a need to find alternatives to synthetic waxes to maintain the quality of horticultural crops such as ‘Kinnow’ mandarin fruit.

Different natural edible coatings (ECs), including gum arabic, carnauba wax (Khorram et al., 2017), chitosan, carboxymethyl cellulose and beeswax (Baswal et al., 2020), have been applied to maintain the quality of ‘Kinnow’ mandarin fruit. Gum arabic, a polysaccharide obtained from the branches and stems of Acacia plants, is a well-known carrier of essential elements such as potassium, calcium, and magnesium (Riva et al., 2020). Its unique characteristics include having a tightly packed hydrogen-bonded network structure, resulting in good oxygen and carbon dioxide barrier properties (Khorram et al., 2017). Additionally, gum arabic ECs can be used as a carrier for active additives such as antioxidants and antibacterial agents and flavors, which are essential elements in maintaining the quality of horticultural crops such as ‘Kinnow’ mandarin fruit. However, gum arabic has been reported to have poor water vapor barrier properties (Khorram et al., 2017; Riva et al., 2020). Due to the restricted use of synthetic fungicides incorporated into ECs, other means of adding antibacterial and antioxidant agents into these ECs must be exploited (La et al., 2021; Nxumalo et al., 2021).

To extend food quality and ensure safety, organic and inorganic materials have been successfully incorporated into ECs (Aloui and Khwaldia, 2016; Nguyen et al., 2020). Organic antimicrobial materials are known to become unstable over an extended period, whereas inorganic materials have exhibited reasonable stability at high temperatures and pressure over time (Sawai, 2003). Inorganic oxides such as magnesium oxide, titanium dioxide, and zinc oxide have been reported as effective antibacterial agents (John Leo and Oluwafemi, 2017), and improved the properties of ECs. These inorganic oxides can be incorporated into an EC matrix (Nxumalo and Fawole, 2022a). Zinc oxide is considered the safest metal oxide for human health because it is one of the essential elements from many metalloenzymes in living organisms, and it is known to have low toxicity; thus, it is used on the linings of food cans, as food additives, and as packaging materials (Stoimenov et al., 2002; John Leo and Oluwafemi, 2017). Therefore, zinc oxide has the potential to be used as an alternative to synthetic fungicides. In a quest to improve the water vapor properties of ECs, nano-sized (1–100 nm) filler materials have been incorporated into the biopolymer coating to create a bio-nanocomposite polymer (Yoksan and Chirachanchai, 2010). According to Slavutsky and Bertuzzi (2014), nanomaterials have been reported to improve the functional properties, structure and steadiness of EC polymer matrix. For example, a study by Arroyo et al. (2020) reported that alginate and chitosan coating enriched with zinc oxide nanoparticles (ZnO-NPs) successfully maintained the quality attributes of guava fruit, while La et al. (2021) observed that ZnO-NPs in chitosan/gum arabic EC matrix maintained the quality and extended the shelf life of banana fruit. Nanocomposite coating based on carrageenan and ZnO-NPs maintained mango quality and extended its shelf life (Meindrawan et al., 2018). Furthermore, the shelf life of strawberry fruit was extended using carboxymethyl cellulose enriched with green synthesized ZnO-NPs (Gao et al., 2020), and similar observations were reported on persimmon and tomato fruit treated with carboxymethyl cellulose coating enriched with ZnO-NPs (Saekow et al., 2019).

The physical and chemical methods currently used to synthesize inorganic metal oxides are associated with a lot of drawbacks to human life and the environment, and they are relatively expensive to use (Mirzaei and Darroudi, 2017; Patil and Taranath, 2018). Based on green chemistry principles, green synthesis of metal oxides is regarded eco-friendly and safe method (Mohan et al., 2014). During green synthesis, plant extracts can be used as reducing, capping, and stabilizing agents of nanoparticles (Mirzaei and Darroudi, 2017; Patil and Taranath, 2018). Green synthesis of nanoparticles is promoted because it is cost-effective, stable, non-hazardous, environmentally benign, and non-toxic (Mittal et al., 2013; Mohan et al., 2014). Indigenous people have been using *Bidens pilosa* L. extracts to treat various ailments (Goudoum et al., 2016; Parveen and Ali, 2019), while its essential oils have been used for conserving and flavoring food (Dong et al., 2004). A study by Nxumalo and Fawole (2022b) revealed that chitosan fused with *B. pilosa* extract maintained the postharvest quality and storage stability of coated purple passion fruit compared to other medicinal plants extracts. This indicates that *B. pilosa* can be used as a green material to synthesize metal oxides.

Many studies have evaluated the application of gum arabic as fruit coating material; however, the utilization of inorganic substances such as green synthesized ZnO-NPs as a filler to enhance the characteristics of gum arabic coatings has not been fully exploited. At present, little is known about the postharvest application of green synthesized ZnO-NPs in gum arabic matrix on the storage life and quality of ‘Kinnow’ mandarin. Therefore, this study evaluated the efficacy of ZnO-NPs in gum arabic matrix in maintaining the quality attributes of mandarin (cv. Kinnow) fruit. This research may contribute to maintaining the storage quality of soft citrus, ‘Kinnow’ mandarin fruit, a major citrus fruit exported from South Africa.

## Materials and Methods

### Procurement and Handling of Medicinal Plants

*Bidens pilosa* plant was used as a biofactory in the green synthesis of ZnO-NPs. The plant material was collected from the Eswatini Institute for Research in Traditional Medicine, Medical and Indigenous Food Plants (EIRMIP) farm at Mafutseni (026°23′S and 031°31′E) in the Kingdom of Eswatini. A taxonomist identified the plant, and a herbarium specimen was archived as KN1001. The leaves were oven dried (Labotec, Bavaria, South Africa) at 50°C for 72 h and ground into a fine powder using a blender (Kambrook, Zhejiang, China) and stored in a ziplock bag until required.

### Extraction of Plant Material and Green Synthesis of Zinc Oxide Nanoparticles

A procedure outlined by Bhuyan et al. (2015) and Santhoshkumar et al. (2017) was adopted with slight modifications. Briefly, 5 g of dried *B. pilosa* leaves were mixed with 100 mL distilled water in a 250 mL Erlenmeyer flask and then boiled at 60°C for 10 min. The mixture was cooled at room temperature, filtered through Whatman No. 1 filter paper, and the filtrate was stored in a refrigerator at 4°C and 90 ± 5% relative humidity (RH) until further use.

Zinc nitrate dihydrate (2 M) was prepared in 100 mL deionized water under constant stirring for 1 h at 80 °C using a hot plate magnetic stirrer, followed by slow addition of 20 mL of 0.05 M NaOH. After 1 h, *B. pilosa* plant extract (25 mL) was slowly added under continuous stirring for 2 h using a magnetic stirrer until a yellow-white precipitate was formed. A pale white solid pellet was collected through centrifugation at 8000 rpm at 30 °C for 15 min, washed three times with distilled water and once with ethanol, and then oven-dried at 100°C for 12 h. The pale white solid material was then placed in an open metal container and heated at 450°C for 4 h using a digitally operated hot plate. The obtained powder material was characterized and stored in an air-tight bottle at room temperature until further use.

### Procurement of ‘Kinnow’ Mandarin Fruit

Matured ‘Kinnow’ mandarins were procured from Casmar Citrus Nursery, Mooinooi, North-West Province, South Africa (-25.77125 E, 27.61348 S). The fruit were transported to the postharvest laboratory, University of Johannesburg, and sorted for blemishes, visible external damage and uniformity of color and size before, disinfected by immersion in 0.04% sodium hypochlorite for 3 min and air-dried using a fan at 25 ± 5°C and 50 ± 5% RH for 30 min.

### Preparation of Coating Solution and Coating Application

In the following order: gum arabic (GA) (2% w/v) was mixed with canola oil (1% v/v), Tween 20 (1% v/v), glycerol (1%) and ZnO-NP (0.25, 0.5 and 1%). A completely randomized design (CRD) was used. ‘Kinnow’ mandarin fruit were divided into five groups, representing each composite coating and the control group. The control treatment was only washed in water containing sodium hypochlorite, while the four groups were further dipped separately in GA, GA + ZnO-NP 0.25%, GA + ZnO-NP 0.5% and GA + ZnO-NP 1% for 3 min, followed by air drying at 20 ± 5°C and 65 ± 5% RH for 1 h. Three standard open-top commercial cartons (35 × 28 cm) containing 12 fruit were used as replicates of each treatment per interval. Fruit were stored at 5 ± 1°C, and 90 ± 5% RH for 40 days and sampling was done at 10 days intervals. To determine the incidence and severity of rind physiological disorders, which manifest under retail conditions, the fruit were further exposed to 20 ± 5°C and 65 ± 5% RH for 5 days.

### Fruit Rind Property Assessment

#### Scanning Electron Microscopy

‘Kinnow’ mandarin rind surface morphology was observed using scanning electron microscopy (SEM, SU8010, Hitachi, Japan) at 10 kV. Rind (10 × 10 mm) were cut, dried, and mounted on aluminum stubs using double-sided carbon tape and sputter-coated with gold. Comparable magnifications of coated and uncoated ‘Kinnow’ rind were imaged (Kawhena et al., 2022).

#### Water Vapor Permeability

Water vapor permeability (WVP) of ‘Kinnow’ mandarin rind was determined gravimetrically (Kritzinger et al., 2018; Salama and Abdel-Aziz, 2021), with slight modifications. The ‘Kinnow’ mandarin rind without defects was cut in a circular shape of 65 mm diameter and mounted on an aluminum permeability cell with an internal diameter opening of 20 mm. The aluminum permeability cell was filled with 25 mL distilled water (100% RH) and maintained at 23°C. Analysis was performed in triplicates. Weight reduction was determined every 2 h for 10 h and thereafter after 24 h. WVP was calculated by weight loss per unit time (*W/t*), rind thickness (*x*), and pressure gradient (Δ*P*), according to equation (1) and expressed in g⋅mm/m^–2^ h^–1^ Pa^–1^:


(1) $$W\,V\,P=(\left[\frac{w}{t}\right]\times X)\,/\,\Delta \,P$$

### Physiological Properties

#### Weight Loss

Weight loss of ‘Kinnow’ mandarin fruit was determined by monitoring weight change in fruit at different intervals using an electronic scale (Mettler Toledo, Model ML3002E, Switzerland, 0.0001 g accuracy). Results were expressed as the percentage weight loss of the initial weight (0 day). Twelve fruits per treatment were evaluated, and results were calculated using equation 2:


(2) $$W=\frac{W_{i}-W_{f}}{W_{i}}\times 100$$

Where *W* = weight loss (%) of fruit; *W*_*i*_ = initial weight (g) of the fruit at the beginning of storage, and *W*_*f*_ = final weight (g) of the fruit at the time of sampling.

#### Respiration Rate

Fruit respiration rate was measured using the closed system method previously described by Caleb et al. (2012) and Fawole et al. (2020). Briefly, in triplicates, five ‘Kinnow’ mandarin fruit were placed in 600 mL containers hermetically sealed with a lid containing a rubber septum in the middle for 2 h at room temperature. After the incubation time, CO_2_ produced inside the container was measured using an infrared O_2_/CO_2_ gas analyser with an accuracy of ± 0.5% (Checkmate 3, PBI Dansensor, Ringstead, Denmark).

### Physical Attributes

#### Rind Color

‘Kinnow’ mandarin fruit citrus color index (CCI) was measured in the CIE L*, a*, b* coordinates using a calibrated Minolta Chroma Meter (Model CR-400, Minolta Corp, Osaka, Japan). On five fruits per treatment, rind color was measured on opposite sides of the equatorial region of each fruit at three marked spots. The CCI was calculated as described by Mendoza et al. (2006) using equation 3:


(3) $$C\,C\,I=\frac{1000\times a}{L\times b}$$

#### Fruit Texture

Fruit texture was determined according to a procedure outlined by Fawole and Opara (2013), adopted with slight modifications. The fruit compression test was performed using a texture analyzer meter (Agrosta texture analyzer, Calib, France) with a 35 mm compression probe. A total of five fruits per treatment were used, and the test was run per fruit aligned horizontally on the compression platform. Textural profile was interpreted using force (N) and distance (mm) as the fundamental variables. The instrument’s operating conditions were: test speed 10 mm/s, post-test speed 50 mm/s and pull or push triggered force of 5 N.

#### Rind Weight

The fruit rind weight was calculated using equation 4:


(4) Rindweight(%)=(average⁢peel⁢weight/average⁢fruit⁢weight)×100

#### Juice Weight

The fruit juice weight was calculated using equation 5:


(5) Juiceweight(%)=(average⁢juice⁢weight/average⁢fruit⁢weight)×100

#### Rind to Pulp Ratio

The fruit rind to pulp ratio was calculated using equation 6:


(6) Rind⁢to⁢pulp⁢ratio=(average⁢rind⁢weight/average⁢juice⁢weight)×100

### Chemical Attributes

#### Rind Electrolyte Leakage

In triplicates, six disks of rind tissue were cut out using a stainless-steel 10 mm cork borer to determine the electrolyte leakage as outlined by Mirdehghan et al. (2007), with slight modifications. The cut discs were then immersed into 20 mL of deionized water and then incubated for 1 h at room temperature (25 ± 5°C and 65 ± 5% RH) under constant shaking for initial data reading (L_t_) using a conductivity meter (Hanna Instruments 9033, Woonsocket, RI, United States). Thereafter, samples were boiled for 15 min in a water bath, and the final data reading (Lo) was taken after it cooled down. Electrolyte leakage was computed as a per cent using equation 7:


(7) $$\mathrm{Rind}\,\mathrm{electrolyte}\,\mathrm{leakage}=\frac{{\text{L}}_{\text{t}}}{{\text{L}}_{\text{o}}}\times 100$$

#### Total Soluble Solids, Titratable Acidity, and BrimA Index

Total soluble solids (TSS) and titratable acidity (TA) were determined from ‘Kinnow’ mandarin juice extracted from 12 fruits per treatment per sampling point. Juice from each fruit was extracted using a juice extractor (Salton juice extractor, Brunswick, NB, Canada). TSS (°Brix) was determined in triplicates using a digital refractometer (Atago, Tokyo, Japan) initially calibrated with distilled water. Titratable acidity (TA%) was determined using an automated Orion Star T940 titrator (Thermo Scientific, Waltham, MA, United States). Briefly, pooled juice samples of five fruits per replicate (triplicate per treatment) were measured by diluting 2 mL of fresh juice with 90 mL of distilled water and titrated with 0.1M NaOH to an endpoint of pH 8.2. BrimA index, a criterion for consumer acceptance of citrus juice, was calculated and expressed as outlined by Magwaza and Opara (2015) using equation 8:


(8) BrimA=TSS-k×TA

Where k is the tongue’s sensitivity index was calculated to be 5 (*k = 5* for citrus fruits).

### Phytochemical Content and Antioxidant Capacity

#### Total Phenolic Content

Total phenolic content (TPC) of ‘Kinnow’ mandarin fruit juice was determined using the Folin-Ciocalteu (Sigma-Aldrich, St. Louis, MO, United States) reagent method according to Siripatrawan and Harte (2010), using gallic acid as a standard with slight modifications. Briefly, juice (1 mL) was extracted with 9 mL of 50% aqueous methanol, and the resulting mixture was vortexed and sonicated in ice for 20 min in an ice bath. ‘Kinnow’ mandarin fruit juice (50 μL) was mixed with 450 μL of 50% methanol followed by 500 μL Folin-C and sodium carbonate (2%) after 2 min. The mixture was vortexed and incubated for 40 min in a dark room. Absorbance measured was read at 725 nm using a UV–visible spectrophotometer (United scientific, SP-UV 300, Johannesburg, South Africa). Gallic acid (Sigma-Aldrich, St. Louis, MO, United States) was used to prepare a calibration curve. The results were expressed as milligrams of gallic acid equivalent per 100 mL of crude juice (mg GAE/100 mL juice).

#### Total Flavonoid Content

Total flavonoid (TFC) was measured using the colorimetric assay outlined by Fawole et al. (2020), with a slight modification. In a test tube, distilled water (4 mL) was added to 1 mL of juice extract, followed by 0.3 mL of 5% sodium nitrite solution and 0.3 mL of 10% aluminum chloride solution. The mixture was incubated at ambient temperature for 5 min before adding 2 mL of 1 M sodium hydroxide. Absorbance was measured using a UV-visible spectrophotometer at 510 nm, and results were expressed as catechin equivalents per 100 mL of crude juice (CAE mg/100 mL juice).

#### Ascorbic Acid Content

Ascorbic acid content was determined using a method described by Fawole et al. (2020) with slight modifications. Briefly, juice (0.5 mL) was extracted with 14.5 mL of 1% metaphosphoric acid (MPA). The extracts (1 mL) were mixed with 9 mL of 2,6-dichlorophenolindophenol (dye) and incubated for 30 min in the dark. The mixture was then vortexed and sonicated (Labotec, Bavaria, South Africa) for 3 min on ice, followed by centrifugation at 4°C for 10 min at 5,000 rpm. Absorbance was measured at 515 nm using a UV-visible spectrophotometer, and ascorbic acid content was calculated based on the calibration curve of standard L-ascorbic acid content. Results were expressed in milligrams of ascorbic acid equivalent per 100 mL of crude juice (mg AAE/100 mL juice).

#### Radical Scavenging Antioxidant Activity

Radical scavenging activity of total antioxidant was determined using the 2,2-diphenyl-1- picryl-hidrazil (DPPH) method based on quantifying the free radical scavenging activity of the juice as described by Siripatrawan and Harte (2010) with slight modifications. Briefly, methanolic juice extract (15 μL) was diluted with 735 μL methanolic DPPH solution (0.1 mM). The mixture was vortexed for 1 min and incubated for 30 min in the dark before measuring the absorbance at 517 nm using a UV-vis spectrophotometer. The free-radical capacity of juice was expressed as ascorbic acid (mM) equivalent per mL juice (mM AAE/mL juice).

#### Ferric Reducing Antioxidant Power

The Ferric Reducing Antioxidant Power (FRAP) antioxidant power of juice was measured according to Fawole et al. (2012) with slight modifications. A FRAP working solution of 300 mM acetate buffer (50 mL), 2,4,6-tripyridyl-s-triazine (TPTZ) (5 mL) and 20 mM FeCl_3_ (5 mL) was freshly prepared prior to the measurements. Diluted aqueous methanolic juice extracts (150 μL) were mixed with 2850 μL of the FRAP working solution before incubation in the dark for 30 min. Thereafter, the absorbance was measured at 593 nm using a UV-vis spectrophotometer. Results were expressed as Trolox (mM) equivalents per mL juice (mM TE/mL juice).

#### ABTS^+^ Radical Scavenging Activity

The ABTS^+^ radical scavenging activity was analyzed according to Tagliazucchia et al. (2010) and Fawole and Opara (2016) with slight modifications. Briefly, the ABTS^+^ (2,2′-azino-bis [3-ethylbenzothiazoline-6-sulphonic acid]) solution containing 7.4 mM ABTS^+^ and 2.6 mM of potassium persulfate was prepared and allowed to stand for 12 h at room temperature in the darkroom to create a stable, dark blue-green radical solution. The working solution was then diluted with methanol to an absorbance of 1.1 ± 0.02 at 734 nm to form the test reagent. Diluted test samples (75 μL) were mixed with 1425 μL of the prepared test reagent and vortexed for 1 min before being incubated for 30 min at room temperature in the dark. Absorbance was measured using a UV-vis spectrophotometer at 734 nm.

### Enzyme Activity Assays

#### Catalase Activity

Catalase (CAT) activity procedure was adopted by measuring the initial rate of increase at 240 nm as outlined by Meighani et al. (2014) and He et al. (2020), with slight modifications. Briefly, crude enzyme extraction was obtained by mixing 2 g of ‘Kinnow’ mandarin rind (KMR) extract with 10 mL of phosphate buffer (pH 7) containing polyvinyl pyrrolidone (PVPP) and ETDA and vortexed for 30 s. The sample was then sonicated in ice at 0°C for 10 min and then centrifuged at 10000 rpm for 15 min at 4°C to obtain the supernatant. Thereafter, 100 μL of enzyme extract, 500 μL of 20 mM H_2_O_2_ and 2.4 mL of phosphate buffer (pH 5) were added into a cuvette. Absorbance was repeatedly measured every 1 min for 3 min using a UV-vis spectrophotometer at 240 nm. An increase in absorbance of 0.01 per min represents 1 enzyme unit. Results were expressed as enzyme unit (U) mL^–1^ min^–1^ dry weight (DW) per KMR and calculated using equation 9:


(9) $$\mathrm{CAT}\,\mathrm{activity}\,U\,{\mathrm{mL}}^{-1}\,{\text{min}}^{-1}\,\mathrm{DW}\,\mathrm{KMR}=\frac{\left({\text{Abs}}_{f}-{\text{Abs}}_{i}\right)\times \mathrm{total}\mathrm{reaction}\mathrm{vol}.}{\mathrm{time}\,\mathrm{interval}\times \mathrm{volume}\,\mathrm{of}\,\mathrm{enzyme}}$$

Where: *Abs*_*f*_ = final absorbance; *Abs*_*i*_ = initial absorbance; total reaction vol. = 3; time interval = 3; volume of enzyme = 0.1 mL.

#### Phenylalanine Ammonia-Lyase Activity

Phenylalanine ammonia-lyase (PAL) activity was determined by measuring the initial rate of increase at 290 nm as outlined by He et al. (2020) and Medda et al. (2020) with slight modifications. Briefly, crude enzyme extraction was obtained by mixing 2 g of KMR with 10 mL of phosphate buffer (pH 7) containing polyvinyl pyrrolidone (PVPP) and ETDA and vortexed for 30 s. The sample was then sonicated in ice at 0°C for 10 min and then centrifuged at 10,000 rpm for 15 min at 4°C to obtain the supernatant. Thereafter, 1 mL of enzyme extract was added to 3 mL of boric acid buffer (in a 30°C water bath for 5 min) and well mixed. Absorbance was measured every 1 min for 3 min at 290 nm against a blank (without enzyme extract). An increase in absorbance of 0.01 per min represents 1 enzyme unit, and it was calculated using equation 10:


(10) $$\mathrm{PAL}\,\mathrm{activity}\,U\,{\mathrm{mL}}^{-1\,}\,{\text{min}}^{-1}\,\mathrm{DW}\,\mathrm{KMR}=\frac{\left({\text{Abs}}_{f}-{\text{Abs}}_{i}\right)\times \mathrm{total}\mathrm{reaction}\mathrm{vol}.}{\mathrm{time}\,\mathrm{interval}\times \mathrm{volume}\,\mathrm{of}\,\mathrm{enzyme}}$$

Where: *Abs*_*f*_ = final absorbance; *Abs*_*i*_ = initial absorbance; total reaction vol. = 4; time interval = 3; volume of enzyme = 1 mL.

#### Peroxidase Activity

Peroxidase (POD) activity was determined by measuring the initial rate of increase at 470 nm as outlined by González et al. (1999) and Meighani et al. (2014) with slight modifications. Briefly, crude enzyme extraction was obtained by mixing 2 g of KMR with 10 mL of phosphate buffer (pH 7) containing polyvinyl pyrrolidone (PVPP) and ETDA and vortexed for 30 s. The sample was then sonicated in ice at 0°C for 10 min followed by centrifugation at 10,000 rpm for 15 min at 4°C to obtain the supernatant. POD activity was determined by adding 200 μL of enzyme extract to 2.2 mL of 0.3% guaiacol in phosphate buffer at 30°C for 5 min and then adding 0.6 mL of 0.3% hydrogen peroxide at 30°C. Absorbance was measured at 470 nm using a UV-vis spectrophotometer every 1 min for 3 min. An increase in absorbance of 0.01 per min represented 1 enzyme unit was calculated as shown in equation 11.


(11) $$\mathrm{POD}\,\mathrm{activity}\,U\,{\mathrm{mL}}^{-1}\,{\text{min}}^{-1\,}\,\mathrm{DW}\,\mathrm{KMR}=\frac{\left({\text{Abs}}_{f}-{\text{Abs}}_{i}\right)\times \mathrm{total}\mathrm{reaction}\mathrm{vol}.}{\mathrm{time}\,\mathrm{interval}\times \mathrm{volume}\,\mathrm{of}\,\mathrm{enzyme}}$$

Where: *Abs*_*f*_ = final absorbance; *Abs*_*i*_ = initial absorbance; total reaction vol. = 3; time interval = 3; volume of enzyme = 0.2 mL.

#### Superoxide Dismutase Activity

Superoxide dismutase (SOD) activity was determined by measuring the initial rate of increase at 560 nm as outlined by He et al. (2020) and Stephenie et al. (2020), adopted with slight modifications. Briefly, crude enzyme extraction was obtained by mixing 2 g of KMR with 10 mL of phosphate buffer (pH 7) containing polyvinyl pyrrolidone (PVPP) and ETDA and vortexed for 30 s. The sample was then sonicated in ice at 0°C for 10 min, followed by centrifugation at 10000 rpm for 15 min at 4°C to obtain the supernatant. Phosphate buffer (1 mL, pH 5), distilled water (1 mL), methionine (300 μL of 22 μM), and nitroblue tetrazolium (NBT; 100 μL of 20 μM) were added to 100 μL of enzyme extract in a cuvette. The cuvette was then placed under UV light for 15 min before 100 μL of 0.6 μM riboflavin (as a substrate) was added to the reaction mixture. Absorbance was measured every 1 min for 3 min at 560 nm using a UV-vis spectrophotometer. One unit of SOD activity was defined as the amount of enzyme required to cause a 50% inhibition of the reduction of NBT as monitored at 560 nm. An increase in absorbance of 0.01 per min represented 1 enzyme unit was calculated as shown in equation (12).


(12) $$\mathrm{SOD}\,\mathrm{activity}\,U\,{\mathrm{mL}}^{-1}\,{\text{min}}^{-1\,}\,\mathrm{DW}\,\mathrm{KMR}=\frac{\left({\text{Abs}}_{f}-{\text{Abs}}_{i}\right)\times \mathrm{total}\mathrm{reaction}\mathrm{vol}.}{\mathrm{time}\,\mathrm{interval}\times \mathrm{volume}\,\mathrm{of}\,\mathrm{enzyme}}$$

Where: *Abs*_*f*_ = final absorbance; *Abs*_*i*_ = initial absorbance; total reaction vol. = 3; time interval = 3; volume of enzyme = 0.1 mL.

### Physiological Disorders

#### Fruit Physiological Disorders

The disorders assessed included rind pitting and chilling injury. These were visually inspected after storing the fruit at room temperature (20 ± 5°C and 65 ± 5% RH) to simulated retail conditions for 5 days. The degree of incidence of the disorders was subjectively determined using a hedonic scale as described by Fawole and Opara (2013), where 0 = none (no symptoms), 1 = trace (1–25%), 2 = slight (26–50%), 3 = moderate (51–75%) and 4 and above = severe (76–100%). The disorder index was then calculated using equation 13:


(13) $$\mathrm{Disorder}\,\mathrm{index}=\frac{\mathrm{value}\,\mathrm{of}\,\mathrm{hedonic}\,\mathrm{scale}\times \mathrm{number}\,\mathrm{of}\,\mathrm{fruit}\,\mathrm{at}\,\mathrm{each}\,\mathrm{scale}}{\mathrm{total}\,\mathrm{number}\,\mathrm{of}\,\mathrm{fruit}}\times 100$$

### Statistical Analysis

Statistical analysis was conducted using GenStat Statistical Software (GenStat, 18.2 edition, VSN International, United Kingdom). Data were subjected to factorial analysis of variance (ANOVA) at a 95% confidence interval. Observed differences at *p* < 0.05 were considered statistically significant according to Duncan’s multiple range test. Mean (±S.E) values of all the studied variables were presented. Pearson linear correlation coefficients (*r*) and principal component analysis (PCA) were carried out using XLSTAT software version 2020.4.1.1020 (Addinsoft, Paris, France).

## Results and Discussion

### Fruit Rind Property Assessment

#### Scanning Electron Microscopy

**FIGURE 1:**
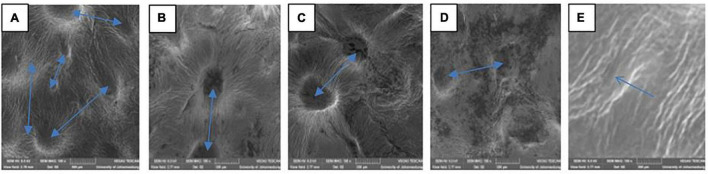
Scanning electron microscopy (SEM MAG: 200×) of ‘Kinnow’ mandarin rinds coated with gum arabic coating fused with zinc oxide nanoparticles. **(A–E)** Correspond to cross-sectional morphology of control; gum arabic coating (2%); gum arabic + zinc oxide nanoparticles (0.25%); gum arabic + zinc oxide nanoparticles (0.5%) and gum arabic + zinc oxide nanoparticles (1%), respectively.

The variation in the fruit membrane image of control and fruit treated with gum arabic (2%) fused with ZnO-NPs were viewed under SEM (Figure 1). SEM is used to beam microstructure and estimate the porosity and pore size distribution of a membrane material (Mulder, 1996). The lenticel opening and distribution of the ‘Kinnow’ mandarin rind were influenced by the coating applied and the incorporated ZnO-NPs in the gum arabic matrix, as shown by the arrows in Figure 1. When viewed under SEM, rind from untreated fruit had large and more visible lenticel openings (Figure 1A). Gum arabic coating alone reduced the lenticel openings (Figure 1B). The gum arabic enriched ZnO-NPs matrix further reduced the openings and their visibility on ‘Kinnow’ mandarin rind, and lenticel openings were reduced with an increase in the concentration of ZnO-NPs in GA coating (Figures 1C–E). These observations agree with Baswal et al. (2020). The authors reported that ‘Kinnow’ mandarin fruit coated with carboxymethyl cellulose and beeswax reduced the lenticel openings on the surface of the fruit, thus, resulting in minimum water loss after 30 days of cold storage. Therefore, from the obtained results, it is logical to suggest that the ZnO-NPs in the gum arabic matrix would influence respiration and transpiration in the investigated fruit, potentially leading to reduced weight loss and maintained fruit quality during storage.

#### Water Vapor Permeability

**FIGURE 2:**
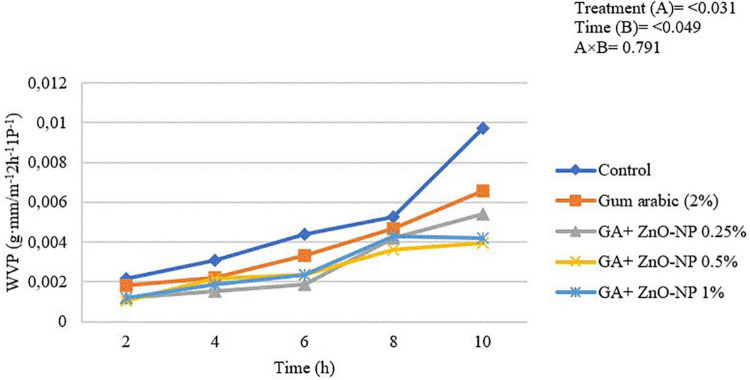
Water vapor permeability of ‘Kinnow’ mandarin rinds for 10 h. Error bars denote the standard error (SE) of the mean. Means ± standard errors presented. To determine the interaction effects, factorial ANOVA was performed for the main factors, treatment, and time. GA + ZnO-NP, gum arabic + zinc oxide nanoparticles; WVP, water vapor permeability.

Edible coatings must have low barrier properties to gaseous exchange and water from food to its surrounding environment (Salama and Abdel-Aziz, 2021). The WVP of the ‘Kinnow’ mandarin rind was significantly (*p* < 0.031) influenced by the treatments applied and the incubation time (*p* < 0.049) (Figure 2). Generally, the WVP of the rinds increased with an increase in the incubation period. The highest WVP (974 × 10^–5^ g⋅mm/m^–2^ h^–1^ Pa^–1^) was observed in control fruit, followed by gum arabic coating alone (657 × 10^–5^ g mm/m^–2^ h^–1^ Pa^–1^), GA + ZnO-NP 0.25% (543 × 10^–5^ g⋅mm/m^–2^ h^–1^ Pa^–1^), GA + ZnO-NP 1% (420 × 10^–5^ g⋅mm/m^–2^ h^–1^ Pa^–1^) and the lowest WVP (397 × 10^–5^ g⋅mm/m^–2^ h^–1^ Pa^–1^) was observed in ‘Kinnow’ mandarin rinds coated with GA+ZnO-NP 0.5%. The decrease in WVP of the ‘Kinnow’ mandarin rind after coating with gum arabic may be attributed to reduced hydrophilic groups between the coating and water molecules (Khoshgozaran-Abras et al., 2012). Therefore, incorporating the ZnO-NPs into the gum arabic matrix further improved the barrier properties of the coating, improving the crosslink interactions between gum arabic bonds, thereby reducing the availability of gum arabic hydrophilic groups.

### Physiological Responses

#### Weight Loss and Respiration Rate

**FIGURE 3:**
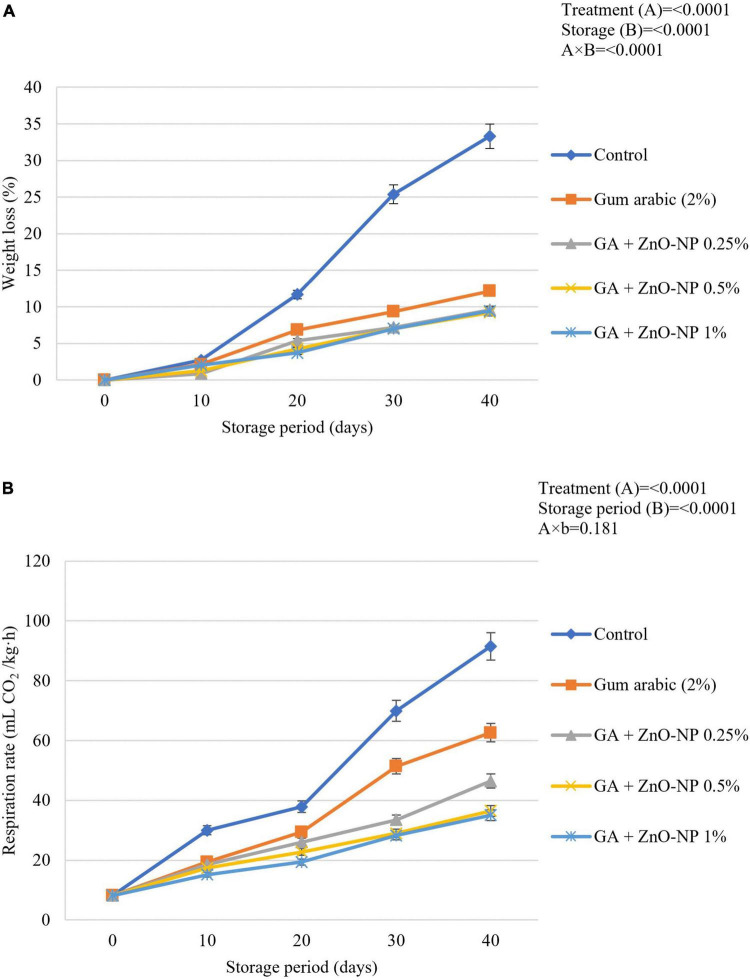
**(A)** Changes in weight loss and **(B)** respiration rate of ‘Kinnow’ mandarin fruit during storage for 40 days at 5 ± 1°C. Error bars denote the standard error (SE) of the mean. Means ± standard errors presented. To determine the interaction effects, factorial ANOVA was performed for the main factors, treatment, and storage time. GA + ZnO-NP, gum arabic + zinc oxide nanoparticles.

Loss of fruit weight during prolonged storage is related to increased respiration rate. ECs such as gum arabic reduce the respiration rate by forming a semi-permeable layer on fruit surfaces, which become a protective barrier against increased respiration processes through the epidermal cells of the fruit (Kumar et al., 2017; Kritzinger et al., 2018). Generally, the postharvest application of ZnO-NPs into the gum arabic matrix significantly (*p* < 0.05) maintained higher fruit weight during the storage compared to control fruit (Figure 3A). It was observed that the interaction between the treatments applied and the storage period significantly (*p* < 0.0001) affected the weight loss of ‘Kinnow’ mandarin fruit. Notably, gum arabic coating enriched with ZnO-NPs significantly (*p* < 0.05) reduced the ‘Kinnow’ mandarin fruit weight loss compared to gum arabic EC alone. However, no significant differences (*p* > 0.05) were observed between the applied ZnO-NPs concentrations in the gum arabic matrix. The highest weight loss was observed in the control fruit (33.3%), followed by gum arabic alone (12.1%), GA + ZnO-NP 0.25% (9.6%), GA + ZnO-NP 1% (9.4%), and the lowest weight loss (9.2%) was observed in fruit treated with GA + ZnO-NP 0.5%. It can be hypothesized that incorporating the ZnO-NPs into the gum arabic matrix improved the water vapor barrier properties and antimicrobial properties of the coating by preventing reactive oxygen species (ROS) (Kritzinger et al., 2018; Riva et al., 2020) and loss of carbon atoms caused by increased metabolism process, thus, resulting in high membrane integrity, of the ‘Kinnow’ mandarin fruit (La et al., 2021). This is in line with the notion that ECs form a barrier to water loss and gas (CO_2_ and O_2_) exchange, thus maintaining the quality of the fruit (Kumar et al., 2017). Similar results were reported by La et al. (2021), where chitosan/gum arabic incorporated with ZnO-NPs significantly lowered the weight loss of banana fruit compared to chitosan and gum arabic alone. Ali et al. (2021) also reported that carboxymethyl cellulose coating (1%) controlled the increase in weight loss of ‘Kinnow’ mandarin better than the control treatment.

Respiration rate is an important indicator of metabolic activity and provides an initial indication of the possible shelf life of stored horticultural crops (Win et al., 2006). A spontaneous increase in respiration rate in both coated and uncoated fruit will prompt sudden multifaceted biochemical changes resulting in a faster ripening rate of fruit. The rate of respiration results in increased metabolic processes and the release of energy and heat, known as latent heat (Wang et al., 2013). Therefore, the respiration rate must be kept as low as possible to maintain the quality and extend the shelf life of horticultural crops such as ‘Kinnow’ mandarin fruit. Results indicated that the respiration rate was driven by the treatments applied (*p* < 0.0001) and the storage duration (*p* < 0.0001). The respiration rate continuously increased during the storage period across all the treatments (Figure 3B). Increasing the concentration of ZnO-NPs in the gum arabic matrix resulted in a reduced respiration rate. Control fruit had the highest respiration rate (91.5 mL CO_2_/kg⋅h), followed by gum arabic coating alone (62.6 mL CO_2_/kg⋅h), GA + ZnO-NP 0.25% (46.8 mL CO_2_/kg⋅h), GA + ZnO-NP 0.5% (36.5 mL CO_2_/kg⋅h) and the lowest respiration rate (35.1 mL CO_2_/kg⋅h) was observed on ‘Kinnow’ mandarin fruit coated with GA + ZnO-NP 1%. ECs can significantly modify the internal gas concentration on coated fruit by providing a gas barrier to CO_2_ and O_2_ exchange (Riva et al., 2020); thus, the coated ‘Kinnow’ mandarin fruit had a lower respiration rate compared to the control treatment. The enhanced effectiveness of ZnO-NPs in gum arabic matrix in reducing the respiration rate compared to gum arabic coating alone could be attributed to the composite coating being more efficient in covering the lenticels and restricting gaseous exchange due to enhanced gas permeability properties and higher membrane integrity. These results are supported by the observed SEM (Figure 1) and WVP results (Figure 2). Similar results were observed by Meindrawan et al. (2018), who reported that coating mango with carrageenan ECs enriched with ZnO-NPs significantly controlled the respiration rate and weight loss better than carrageenan ECs alone and control treatment. According to Duncan (2011), the filler of ZnO-NPs in ECs enhances the polymer’s barrier properties by tortuous pathway.

### Physical Attributes

#### Color Attributes

**FIGURE 4:**
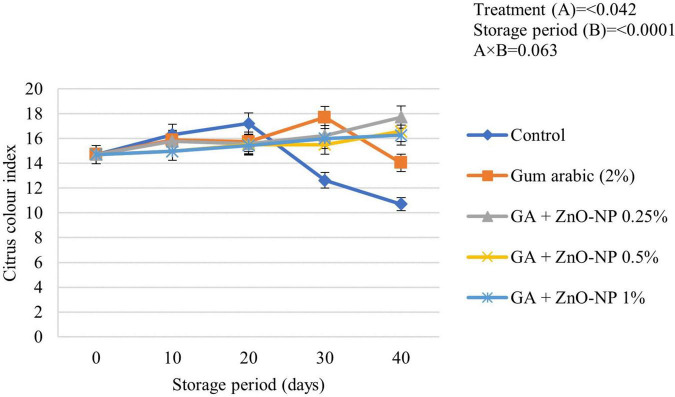
Citrus color index of ‘Kinnow’ mandarin fruit during storage for 40 days at 5 ± 1°C. Error bars denote the standard error (SE) of the mean. Means ± standard errors presented. To determine the interaction effects, factorial ANOVA was performed for the main factors, treatment, and storage time. GA + ZnO-NP, gum arabic + zinc oxide nanoparticles.

Color is an important factor in the consumer acceptance of fruit quality. Citrus fruits are harvested at physiological maturity; therefore, citrus color index (CCI) changes during storage (Mendoza et al., 2006). Some citrus fruits are subjected to certain de-greening treatments, depending on their standard CCI at harvest (Vidal et al., 2012). Generally, the CCI of ‘Kinnow’ mandarin fruit was significantly driven by the treatments applied (*p* < 0.042) and the storage period (*p* < 0.0001) (Figure 4). Control fruit exhibited a rapid increase in CCI and attained the highest peak (17.21) after 20 days of storage before an eventual decline. Gum arabic coating alone showed a steady increase in the CCI and reached a peak of (17.69) before decreasing after 30 days. ‘Kinnow’ mandarin fruit coated with the gum arabic enriched with ZnO-NPs at different concentrations showed a steady increase in CCI value during the 40 days of storage. The respiration rate, as well as the occurrence and severity of postharvest physiological disorders such as rind pitting and chilling injury on the surface of the fruit, influence the CCI of citrus fruit (Santos et al., 2021); thus, this might have contributed to the observed CCI values in this study.

#### Fruit Texture

**FIGURE 5:**
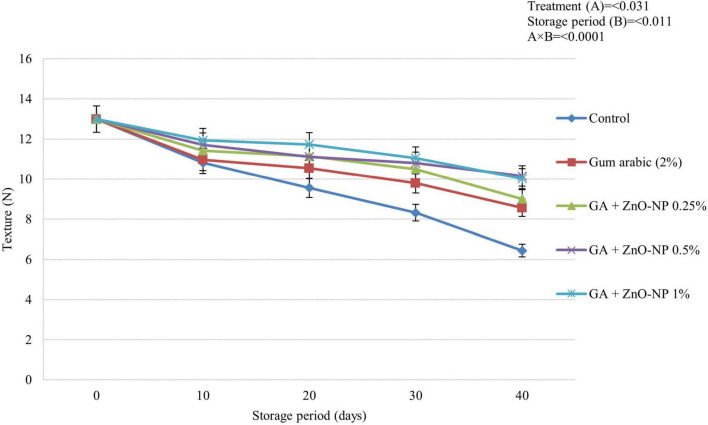
The texture of ‘Kinnow’ mandarin fruit during storage for 40 days at 5 ± 1°C. Error bars denote the standard error (SE) of the mean. Means ± standard errors presented. To determine the interaction effects, factorial ANOVA was performed for the main factors, treatment, and storage time. GA + ZnO-NP, gum arabic + zinc oxide nanoparticles; N, Newtons.

Texture changes during fruit ripening are caused by an alteration in the cell wall structure and sucrose degradation by enzymes that produce simple sugars (Kittur et al., 2001). ECs are known to retain the texture of horticultural crops by decreasing the water vapor transmission rate to reduce water loss. This then inhibits the degradation of insoluble pectin and protopectin (Moalemiyan et al., 2011). Fruit texture was significantly (*p* < 0.0001) influenced by the interaction between the treatments and the storage period (Figure 5). It was observed that the fruit texture significantly (*p* < 0.031) decreased across all the treatments during the storage period; however, it decreased more slowly in treated fruit. After 40 days of storage, ‘Kinnow’ mandarin fruit coated with GA + ZnO-NP 0.5% showed an optimum effect in retaining fruit texture compared to all the treatments. Therefore, the highest fruit texture (10.15 N) was observed on GA + ZnO-NP 0.5% coated fruit, followed by GA + ZnO-NP 1% (10.02 N), GA + ZnO-NP 0.25% (9.01 N), gum arabic coating alone (8.56 N) and the lowest fruit texture (6.44 N) was observed in control fruit. The delay in loss of fruit texture as influenced by both gum arabic coating and the zinc-oxide nanoparticles in gum Arabic matrix may be attributed to a modified fruit atmosphere, resulting in reduced activity of cell wall degradation enzymes such as polygalacturonase, pectin methylesterase, galactosidase, and cellulose (Sayyari et al., 2016). Reduction in these enzyme activity results in increased pectin levels, an essential substance involved in the mechanical strength of the cell wall (Baswal et al., 2020). The formation of ZnO-NPs aggregation in gum arabic matrix may influence the barrier ability of the coating material, resulting in firmer fruit during the storage period.

#### Rind Weight, Juice Weight, and Rind to Pulp Ratio

**FIGURE 6:**
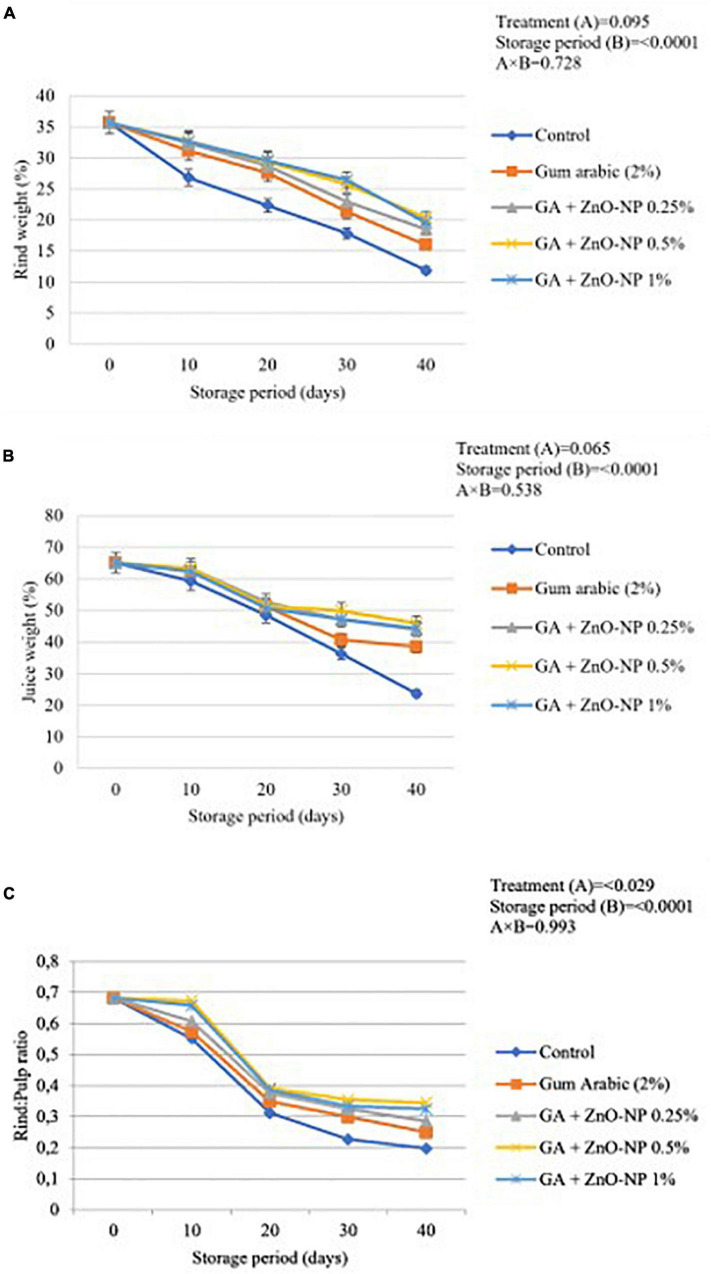
**(A)** Changes in rind weight loss, **(B)** juice weight, and **(C)** rind: pulp ratio of ‘Kinnow’ mandarin fruit during storage for 40 days at 5 ± 1°C. Error bars denote the standard error (SE) of the mean. Means ± standard errors presented. To determine the interaction effects, factorial ANOVA was performed for the main factors, treatment, and storage time. GA + ZnO-NP, gum arabic + zinc-oxide nanoparticles.

Generally, control fruit exhibited significantly reduced rind weight, juice weight percentage and rind to pulp ratio than coated fruit. Results indicated that the rind weight, juice weight and rind to pulp ratio were significantly (*p* < 0.0001) influenced by the storage period (Figure 6). The lowest rind weight, juice weight, and rind to pulp ratio (11.87, 23.69, and 0.197%, respectively) were observed on control fruit, followed by gum arabic coating alone (15.98, 38.49, and 0.249%, respectively), GA+ZnO-NP 0.25% (18.52%, 43.93% and 0.286, respectively), GA + ZnO-NP 1% (19.58, 44.39, and 0.324%, respectively) and the highest rind weight, juice weight and rind to pulp ratio (20.33, 45.94, and 0.345%, respectively) were observed on ‘Kinnow’ mandarin fruit coated with GA+ZnO-NP 0.5%. No significant differences (*p* > 0.05) were observed in the rind and juice weight of ‘Kinnow’ mandarin fruit coated with a composite coating (Figures 6A,B). However, significant differences (*p* < 0.05) were observed in the rind to pulp ratio amongst the ‘Kinnow’ mandarin fruit coated with the different composite coating concentrations (Figure 6C). The rind weight, juice weight and rind to pulp ratio can be attributed to the moisture loss and eventual weight loss of the ‘Kinnow’ mandarin fruit. Similar results were observed by Haider et al. (2020), who reported that applying salicylic acid on ‘Kinnow’ mandarin fruit maintained higher rind weight, juice weight and rind to pulp ratio compared to the control over 90 days of storage.

### Chemical Attributes

#### Rind Electrolyte Leakage

**FIGURE 7:**
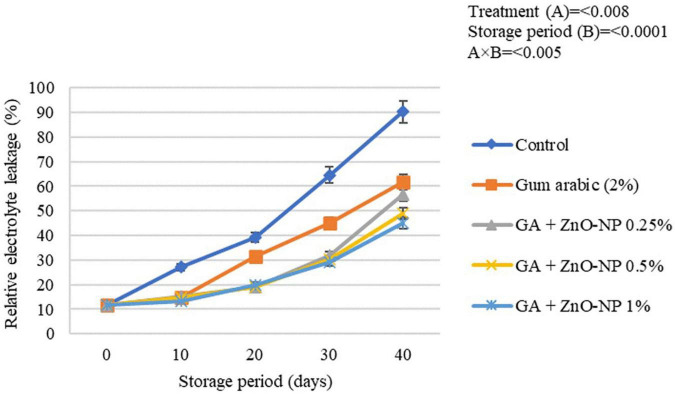
Relative electrolyte leakage of ‘Kinnow’ mandarin fruit during storage for 40 days at 5 ± 1°C. Error bars denote the standard error (SE) of the mean. Means ± standard errors presented. To determine the interaction effects, factorial ANOVA was performed for the main factors, treatment, and storage time. GA + ZnO-NP, gum arabic + zinc oxide nanoparticles.

Generally, electrolyte leakage is regarded as an index that quantifies membrane damage and loss of membrane integrity of fresh produce over time (Sayyari and Ghanbari, 2013). An increase in hydrogen peroxide (H_2_O_2_) and accumulation of reactive oxidation species (ROS) increase the fruit’s oxidative stress, thus, causing an increase in membrane leakage and metabolites and destroying cell membrane in stored fruits. Once the cell membrane is damaged, its permeability increases, causing an increase in the electrolyte leakage rate (Antunes et al., 2010; Shi et al., 2013). Generally, the rind electrolyte leakage of the stored ‘Kinnow’ mandarin fruit increased with the storage period in all the treatments (Figure 7). The increase in rind electrolyte leakage of the ‘Kinnow’ mandarin fruit was influenced by an interaction between the treatments and the storage period (*p* < 0.005). Significant differences (*p* < 0.008) were observed between the control and coated fruit; however, there were no significant differences (*p* > 0.05) among ‘Kinnow’ mandarin fruit coated with gum arabic enriched with ZnO-NPs. The highest rind electrolyte leakage (90.3%) was observed in control fruit, followed by gum arabic coating alone (61.7%), GA + ZnO-NP 0.25% (45.1%), GA + ZnO-NP 1% (44.9%) and the lowest rind electrolyte leakage (43.8%) was observed in GA + ZnO-NP 0.5% coated fruit. The application of gum arabic EC alone delayed loss in cell membrane permeability. However, incorporating ZnO-NPs into the gum arabic matrix may have improved the resistance of the cell membrane to ROS, thus reducing electrolyte leakage in the fruit. Similar results were observed by Ali et al. (2021), who reported that coating ‘Kinnow’ mandarin fruit with carboxymethyl cellulose resulted in 1.49-folds lower rind electrolyte leakage after 30 days in contrast to the control.

#### Total Soluble Solids, Titratable Acidity, and BrimA Index

Changes in TSS, TA, and BrimA index are presented in Table 1. Accumulation of TSS content is one of the important fruit quality indicators of maturity and ripening. TSS content was significantly (*p* < 0.0001) influenced by the interaction between the treatments and the storage period. The initial TSS was 11.7 °Brix, and a steady increase in TSS was observed in the ‘Kinnow’ mandarin fruit across all treatments during the storage period (Table 1). It was observed that the control exhibited higher TSS (14.9 °Brix) during the first 20 days of storage, and thereafter, it started to decline. However, at the end of the storage period, the control fruit exhibited the lowest TSS (10.3°Brix), indicating a higher senescence rate compared with treated fruit. At the end of the storage period, ‘Kinnow’ mandarin fruit coated with gum arabic alone exhibited higher TSS (14.7°Brix), followed by GA + ZnO-NP 0.25% (14.2°Brix), GA + ZnO-NP 1% (13.8°Brix) and the lowest TSS was observed in fruit coated with GA + ZnO-NP 0.5% (13.4°Brix). The increase in TSS could be attributed to conversion of sucrose to sugars (Ali et al., 2010), primarily leading to an increase in TSS concentration during metabolic processes at storage (Cheìour et al., 1990; Parven et al., 2020).

**TABLE 1:** Total soluble solids (TSS), titratable acidity (TA), and BrimA index of ‘Kinnow’ mandarin fruit treated with gum arabic coating fused with zinc-oxide nanoparticles during storage for 40 days at 5 ± 1°C.

Parameter	Treatment	Harvest	Storage period (days)				Significance level		
			10	20	30	40	Treatment (A)	Storage period (B)	A × B
TSS (°Brix)		11.7 ± 0.120							
	Gum arabic (2%)		11.6 ± 0.176^d^	12.5 ± 0.240^cd^	12.9 ± 0.067^c^	14.7 ± 0.133^a^	0.023	<0.0001	<0.0001
	GA + ZnO-NP 0.25%		11.6 ± 0.133^d^	12.4 ± 0.1^cd^	12.1 ± 0.033^cd^	14.2 ± 0.058^ab^			
	GA + ZnO-NP 0.5%		11.8 ± 0.088^d^	12.2 ± 0.067^cd^	12.3 ± 0.033^cd^	13.4 ± 0.318^b^			
	GA + ZnO-NP 1%		12.4 ± 0.319^cd^	13.4 ± 0.058^bc^	13.7 ± 0.133^b^	13.8 ± 0.058^b^			
	Control		12.5 ± 0.121^cd^	14.9 ± 0.033^a^	12.3 ± 0.067^acd^	10.3 ± 0.153^e^			
TA (% citric acid)		2.12 ± 0.005							
	Gum arabic (2%)		1.52 ± 0.047^b^	1.49 ± 0.008^b^	1.37 ± 0.046^c^	1.13 ± 0.078^d^	0.035	<0.0001	<0.0001
	GA + ZnO-NP 0.25%		1.78 ± 0.010^a^	1.70 ± 0.047^a^	1.48 ± 0.008^b^	1.17 ± 0.015^d^			
	GA + ZnO-NP 0.5%		1.71 ± 0.042^a^	1.71 ± 0.042^a^	1.51 ± 0.098^ab^	1.21 ± 0.008^d^			
	GA + ZnO-NP 1%		1.73 ± 0.027^a^	1.36 ± 0.036^c^	1.39 ± 0.016^bc^	1.18 ± 0.012^d^			
	Control		1.36 ± 0.051^c^	1.27 ± 0.076^cd^	1.64 ± 0.040^ab^	1.81 ± 0.127^a^			
BrimA		7.69 ± 0.131							
	Gum arabic (2%)		8.34 ± 0.047^g^	8.49 ± 0.073^f^	10.29 ± 0.125^c^	9.01 ± 0.216^e^	0.039	<0.0001	<0.0001
	GA + ZnO-NP 0.25%		8.32 ± 0.069^g^	8.52 ± 0.074^e^	9.16 ± 0.329^de^	11.87 ± 0.008^a^			
	GA + ZnO-NP 0.5%		8.12 ± 0.254^g^	8.75 ± 0.096^e^	9.97 ± 0.065^d^	11.59 ± 0.003^a^			
	GA + ZnO-NP 1%		8.84 ± 0.152^e^	8.87 ± 0.241^e^	10.03 ± 0.316^cd^	11.61 ± 0.0857^a^			
	Control		10.11 ± 0.069^cd^	10.92 ± 0.277^b^	8.57 ± 0.391^e^	8.10 ± 0.217^g^			

*Data presented as mean ± SE. Different letters across treatments and storage duration for each attribute differ significantly (p < 0.05) according to Duncan’s multiple range test. GA + ZnO-NP, gum arabic + zinc oxide nanoparticles.*

The initial titratable acidity (TA) of the fruit at harvest was 2.12% citric acid. The TA content was significantly (*p* < 0.0001) influenced by the interaction between the treatments applied and the storage period (Table 1). It was observed that coating the ‘Kinnow’ mandarin fruit delayed the degradation of TA in the fruit. As the TSS content increased, the TA content decreased; therefore, the level of TA can be correlated to the accumulation of TSS. The control fruit exhibited a high degradation of TA in the first 20 days (1.27%); however, it increased to 1.81% at the end of the storage period, probably due to a rapid concentration effect of organic acids as a result of higher moisture loss in the fruit. On the other hand, the coated fruit exhibited a steady decline in TA during the storage period. ‘Kinnow’ mandarin fruit coated with gum arabic coating alone had the lowest TA (1.14%), followed by GA + ZnO-NP 0.25% (1.17%), GA + ZnO-NP 1% (1.18%) and the highest TA (1.21%) was observed in fruit coated with GA + ZnO-NP 0.5% after 40 days of storage. The gradual decline in TA of ‘Kinnow’ mandarin fruit with storage indicates the ongoing metabolic processes in the fruit during storage. Organic acids are respiratory substrates in fruits (Sayyari et al., 2011); thus, the slow degradation of TA in coated fruit could be due to a reduced respiration rate in the treated fruit.

BrimA index is based on the TSS/TA ratio and tongue’s sensitivity index (‘*k*’) to determine the acceptability of juices (Jordan et al., 2001). The initial BrimA index was 7.69. The interaction between the treatments and the storage period significantly (*p* < 0.0001) affected the BrimA index of ‘Kinnow’ mandarin fruit (Table 1). Generally, the BrimA index of the fruit significantly (*p* < 0.05) increased during the storage period. The interaction between the storage period and the treatments significantly (*p* < 0.0001) influenced the BrimA index. It was observed that the control fruit exhibited a rapid increase in BrimA index (10.92) compared to the coated fruit. However, after 20 days of storage, BrimA index of the control fruit started to decline. ‘Kinnow’ mandarin fruit coated with gum arabic coating exhibited a steady increase in BrimA index until 30 days of storage before an eventual decline (Table 1). At the end of the storage period, it was observed that GA + ZnO-NP 0.25% exhibited the highest BrimA index (11.87), followed by GA + ZnO-NP 1% (11.61), GA + ZnO-NP 0.5% (11.59), gum arabic coating alone (9.01) and the lowest BrimA index was observed in control fruit (8.10). Similar results were also reported by Nxumalo and Fawole (2022b), who observed an increase in BrimA index with prolonged storage in passion fruit (cv. Ester).

### Phytochemical Analysis

#### Total Phenolic, Total Flavonoid, and Ascorbic Acid Content

Changes in the total phenolic, total flavonoid and ascorbic content in coated and control fruit during storage are presented in Table 2. Generally, TPC significantly (*p* < 0.0001) increased for the first 20 days of storage, followed by a decrease in TPC with a prolonged storage period. Notably, coating ‘Kinnow’ mandarin fruit significantly (*p* < 0.0001) suppressed the rapid decline of TPC during storage. At the end of the storage period (40 days), fruit coated with GA + ZnO-NP 1% exhibited the highest TPC (171.42 mg GAE/100 mL), followed by GA + ZnO-NP 0.5% (168.35 mg GAE/100 mL), GA + ZnO-NP 0.25% (166.87 mg GAE/100 mL), gum arabic alone (146.67 mg GAE/100 mL) and control fruit had the lowest TPC (109.31 mg GAE/100 mL). Similarly, total flavonoid content (TFC) of ‘Kinnow’ mandarin fruit increased for the first 20 days of storage in all treatments before an eventual decline. TFC was significantly (*p* < 0.0001) influenced by the interaction between the treatments applied and the storage duration (Table 2). After 40 days of storage, it was observed that coating ‘Kinnow’ mandarin fruit with GA + ZnO-NP 1% highly suppressed the fast decline of TFC (78.87 mg GAE/100 mL), followed by GA+ZnO-NP 0.5% (73.12 mg GAE/100 mL), GA + ZnO-NP 0.25% (69.15 mg GAE/100 mL), gum arabic alone (58.35 mg GAE/100 mL) and the lowest TFC (46.87 mg GAE/100 mL) was observed in control fruit. Flavonoids are one of the major polyphenols in fruits; hence, the increase in TFC could be associated with the observed increase in TPC (Brouillard et al., 1997). Changes in TPC of fruits during cold storage can be attributed to changes in PAL and polyphenol oxidase (PPO) enzyme activities. The biosynthesis of phenolic compounds through the phenylpropanoid pathway is directly influenced by PAL enzyme activity. On the other hand, PPO enzyme activity oxidizes phenolic compounds using oxygen as a co-substrate (Meighani et al., 2014). Therefore, the higher TPC and TFC in treated fruit could be linked to the reduced PPO activity due to lower oxygen within the coated fruit (Meighani et al., 2014). Fruit coated with gum arabic coating fruits has a layer of film that can regulate the metabolic rate and ROS around the surface of the fruit, thus reducing fast degradation of TPC and TFC in ‘Kinnow’ mandarin fruit. Enriching the gum arabic coating with ZnO-NPs further improved the capacity of the fruit against ROS; thus, the observed higher TPC and TFC in ‘Kinnow’ mandarin fruit coated with gum arabic coatings enriched with ZnO-NPs.

**TABLE 2:** Total phenolic (TPC), total flavonoid (TFC), and ascorbic acid (AA) content of ‘Kinnow’ mandarin fruit treated with gum arabic coating fused with zinc oxide nanoparticles during storage for 40 days at 5 ± 1°C.

Parameter	Treatment	Harvest	Storage period (days)				Significance level		
			10	20	30	40	Treatment (A)	Storage period (B)	A × B
TPC (mg GAE/100 mL KMFJ)		199.577 ± 1.47							
	GA-ZnOP-1%		206.98 ± 1.55^c^	232.27 ± 16.08^ab^	188.04 ± 1.49^d^	171.42 ± 0.59^de^	0.0001	<0.0001	<0.0001
	GA-ZnOP-0.5%		206.13 ± 7.58^c^	245.07 ± 3.34^a^	193.34 ± 2.97^d^	168.35 ± 0.60^e^			
	GA-ZnOP-0.25%		200.74 ± 0.76^c^	241.79 ± 5.49^a^	187.51 ± 4.23^d^	166.87 ± 0.22^e^			
	Gum arabic (2%)		209.62 ± 4.72^bc^	222.01 ± 9.24^b^	184.65 ± 1.97^d^	146.67 ± 5.85^f^			
	Control		204.33 ± 3.54^c^	168.14 ± 4.27^e^	123.38 ± 19.24^g^	109.31 ± 2.66^h^			
TFC (mg CAE/100 mL KMFJ)		77.33 ± 1.06							
	GA-ZnOP-1%		84.65 ± 0.211^a^	88.99 ± 1.22^a^	85.71 ± 0.79^a^	78.87 ± 1.52^b^	<0.0001	<0.0001	0.0001
	GA-ZnOP-0.5%		83.596 ± 1.17^a^	87.41 ± 0.86^a^	85.18 ± 0.38^a^	73.12 ± 0.74^b^			
	GA-ZnOP-0.25%		83.38 ± 0.69^a^	85.39 ± 0.83^a^	80.85 ± 0.27^ab^	69.15 ± 0.58^d^			
	Gum arabic (2%)		78.36 ± 0.04^ab^	84.65 ± 0.27^a^	71.69 ± 1.48^b^	58.35 ± 0.38^f^			
	Control		78.31 ± 0.46^b^	70.58 ± 1.14^c^	64.65 ± 0.90^e^	46.87 ± 0.46^g^			
AA (mg AAE/100 mL KMFJ)		83.93 ± 0.445							
	GA-ZnOP-1%		81.09 ± 0.46^a^	77.03 ± 1.32^bc^	73.87 ± 0.83^c^	63.56 ± 1.27^e^	<0.0001	<0.0001	<0.0001
	GA-ZnOP-0.5%		79.17 ± 0.61^b^	72.38 ± 2.91^c^	69.574 ± 0.83^d^	60.43 ± 1.81^f^			
	GA-ZnOP-0.25%		78.99 ± 1.43^b^	70.16 ± 0.28^cd^	65.79 ± 0.06^e^	56.16 ± 1.35^g^			
	Gum arabic (2%)		71.77 ± 2.78^c^	68.93 ± 1.14^d^	60.59 ± 0.65^f^	50.47 ± 0.52*^h^*			
	Control		70.48 ± 0.66^cd^	56.40 ± 0.81^d^	48.50 ± 0.18^i^	39.73 ± 0.37^j^			

*Data presented as mean ± SE. Different letters across treatments and storage duration for each attribute differ significantly (p < 0.05) according to Duncan’s multiple range test. GA + ZnO-NP, gum arabic + zinc oxide nanoparticles; GAE, gallic acid equivalent; CAE, catechin equivalent; AAE, ascorbic acid equivalent; KMF, ‘Kinnow’ mandarin fruit juice; SE, standard error.*

Like any citrus fruit, ‘Kinnow’ mandarin is a good source of ascorbic acid. Generally, changes in ascorbic acid (AA) content were significantly (*p* < 0.0001) influenced by the interaction between the treatments and the storage period (Table 2). It was observed that ascorbic acid content declined across all the treatments during the storage period. Increasing the concentration of ZnO-NPs in the gum arabic matrix resulted in higher ascorbic acid contents during the 40 days of storage. For example, ‘Kinnow’ mandarin fruit with GA + ZnO-NP 1% had higher ascorbic acid content (63.56 mg AAE/100 mL), followed by GA + ZnO-NP 0.5% (60.43 mg AAE/100 mL), GA + ZnO-NP 0.25% (56.16 mg AAE/100 mL), gum arabic coating alone (50.47 mg AAE/100 mL) and the lowest ascorbic acid content (39.73 mg AAE/100 mL) was observed in the control fruit. The decrease in ascorbic acid content is influenced by oxygen content that can degrade ascorbic acid oxidase and phenoloxidase during storage, thus decreasing the ascorbic acid content in the fruit (Zhou et al., 2008). Furthermore, ascorbic acid breaks down hydrogen peroxide into water (Wang and Gao, 2013), depleting ascorbic acid and promoting physiological disorders and senescence in fruit. It is logical to hypothesize that coating ‘Kinnow’ mandarin fruit with gum arabic resulted in reduced oxygen availability and reduced the extent of oxidation of compounds such as ascorbic acid. Incorporating the ZnO-NPs into the gum arabic matrix further reduced oxidation of ascorbic acid by limiting the generation of free radicals on the surface of ‘Kinnow’ mandarin fruit, thus limiting the degradation of the ascorbic acid content in the fruit.

### Antioxidant Capacity

#### 2,2-Diphenyl-1-Picryl-Hidrazil Radical Scavenging Activity, Ferric Ion Reducing Antioxidant Power, ABTS^+^ Radical Scavenging Activity

**FIGURE 8:**
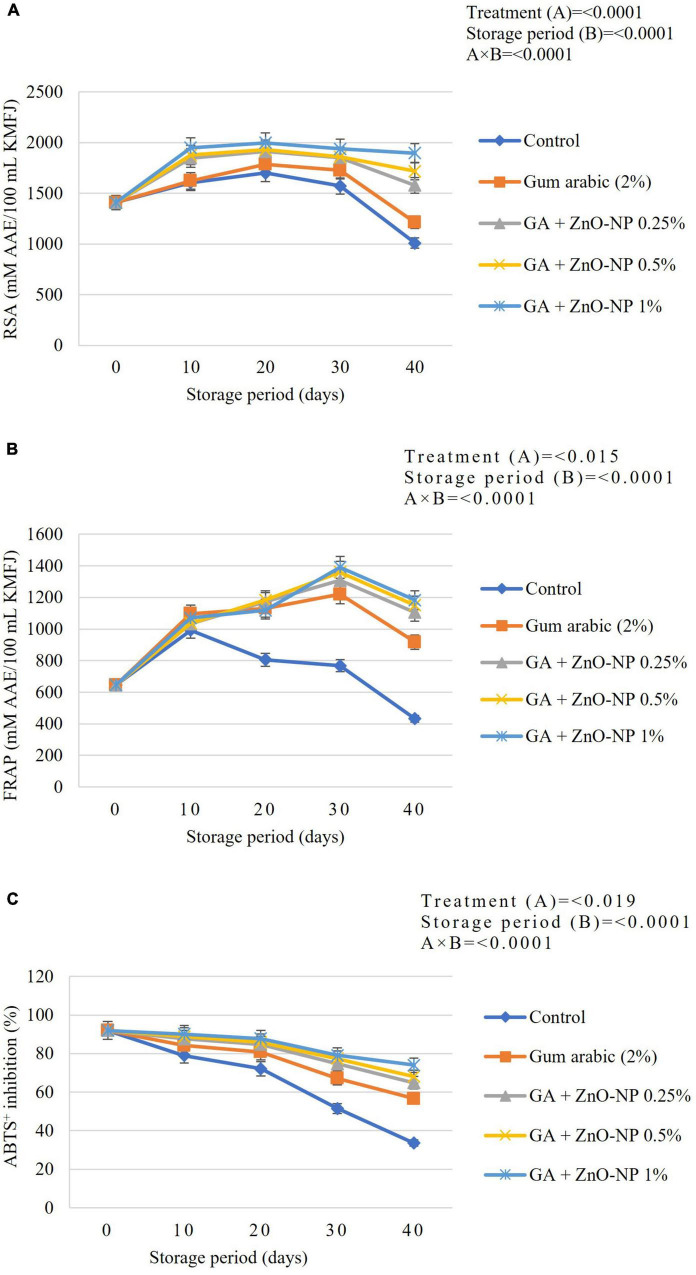
**(A)** Radical scavenging activity (RSA), **(B)** ferric ion reducing antioxidant power (FRAP), and **(C)** ABTS^+^ radical scavenging activity of ‘Kinnow’ mandarin fruit during storage for 40 days at 5 ± 1°C. Error bars denote the standard error (SE) of the mean. Means ± standard errors presented. To determine the interaction effects, factorial ANOVA was performed for the main factors, treatment, and storage time. GA + ZnO-NP, gum arabic + zinc oxide nanoparticles; AAE, ascorbic acid equivalent; TE, trolox equivalent; DW, dried weight; KMFJ, ‘Kinnow’ mandarin fruit juice; SE, standard error.

DPPH radical scavenging activity (RSA) of ‘Kinnow’ mandarin fruit was significantly (p < 0.0001) influenced by the interaction between treatments and the storage period (Figure 8A). The radical scavenging activity of the ‘Kinnow’ mandarin fruit increased in the first 20 days and declined until the end of the storage period in all the treatments. The highest increase in radical scavenging activity (1998 mM AAE/100 mL) was observed in fruit coated with GA + ZnO-NP 1%, followed by GA + ZnO-NP 0.5% (1929 mM AAE/100 mL), GA + ZnO-NP 0.25% (1912 mM AAE/100 mL) and gum arabic coating alone (1787 mM AAE/100 mL), while the lowest peak (1701 mM AAE/100 mL) was observed in control fruit. A similar trend was observed in the radical scavenging activity of ‘Kinnow’ mandarin fruit at the end of the storage period, with ‘Kinnow’ mandarin fruit coated with GA + ZnO-NP 1% maintaining the highest RSA (1897.99 mM AAE/100 mL). This suggests that incorporating the ZnO-NPs into the gum arabic matrix maintained higher RSA. The higher radical scavenging activity of gum arabic enriched with ZnO-NPs may be attributed to the enhanced antioxidant enzyme activity induced by the ZnO-NPs, thereby improving the fruit’s overall antioxidant capacity and scavenging ability (Gao et al., 2020). The coating controlled the oxidation of free radicals, thereby maintaining higher radical scavenging activity compared to uncoated fruit (Matthes and Schmitz-Eiberger, 2009). Bioactive compounds found in ZnO-NPs improved compounds such as ascorbic acid, phenolic and flavonoids (La et al., 2021), thus contributing to RSA of functional additives present in the gum arabic coating.

Antioxidant capacity was also measured using the FRAP method. Results indicated an increase in FRAP activity during the storage period in all the treatments, but it eventually declined as the storage period increased (Figure 8B). Results were influenced by a significant interaction (*p* < 0.0001) between the treatments applied and the storage period. The coated fruit showed a significantly (*p* < 0.0001) higher FRAP activity throughout the storage period than the control fruit. Induced accumulation of antioxidant enzyme activities might contribute to the increase in the antioxidant power of the fruit during the storage period. Antioxidant power increased in the control fruit until 10 days of storage, whereas in the coated fruit, antioxidant power increased until 30 days of storage (Figure 8B). At the end of the storage period, the lowest FRAP activity (432.57 mM AAE/100 mL) was observed in control, followed by gum arabic coating alone (917.48 mM AAE/100 mL), GA + ZnO-NP 0.25% (1105.56 mM AAE/100 mL), GA + ZnO-NP 0.5% (1150.21 mM AAE/100 mL), and the highest FRAP activity (1182.96 mM AAE/100 mL) was observed in GA + ZnO-NP 1% coated fruit. Riva et al. (2020) reported that EC reduces the damage caused by ROS, thereby increasing the antioxidant capacity such as FRAP, thus reducing physiological disorders and fruit decay.

There was a decline in the ABTS^+^ radical scavenging activity of ‘Kinnow’ mandarin fruit across the treatments (Figure 8C). A significant interaction (*p* < 0.0001) between the treatments applied and the storage period was observed in the ABTS^+^ radical scavenging. At the end of the storage period, fruit coated with GA + ZnO-NP 1% had the highest ABTS^+^ radical scavenging (74.03%), followed by GA + ZnO-NP 0.5% (68.11%), GA + ZnO-NP 0.25% (64.89%), gum arabic coating alone (56.59%) and the lowest ABTS^+^ radical scavenging (33.56%) was observed in the control fruit. It was observed that coating the ‘Kinnow’ mandarin fruit significantly (*p* < 0.019) controlled the fast degradation of ABTS^+^ radical scavenging activity of ‘Kinnow’ mandarin fruit during the storage period. Incorporating the ZnO-NPs into the gum arabic matrix further improved the capacity of the coated fruit against ABTS^+^ (Figure 8C). The application of these postharvest treatments affected the metabolic activity of the coated produce by activating the antioxidant system. This activation happens in response to postharvest stress, which is beneficial as it ameliorates the antioxidant potential of tropical fruits (Hong et al., 2012). Therefore, the ABTS^+^ radical scavenging activity in coated fruit could be correlated to maintaining high levels of phytochemicals in the coated fruit (Amorati et al., 2013).

### Antioxidant Enzyme Activity

#### Catalase, Phenylalanine Ammonia-Lyase, Peroxidase, and Superoxide Dismutase Activity

**FIGURE 9:**
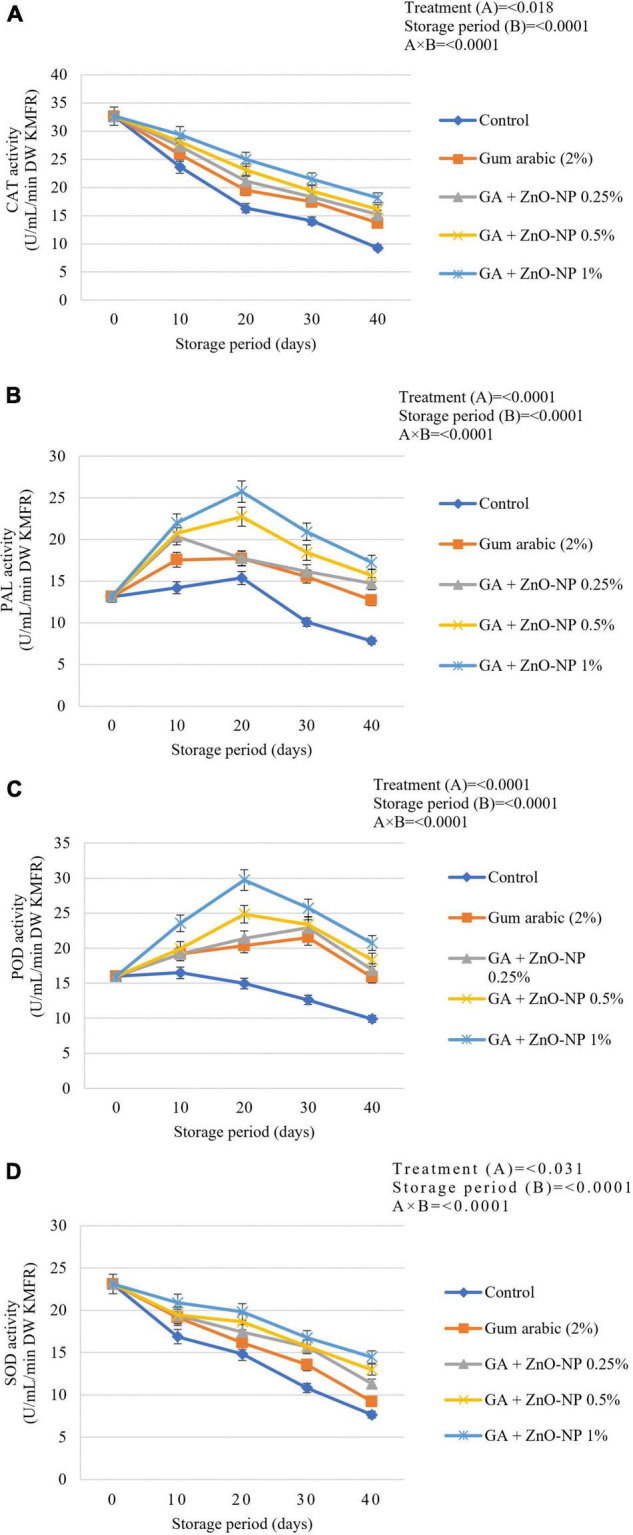
**(A)** Catalase (CAT), **(B)** phenylalanine ammonia-lyase (PAL), **(C)** peroxidase (POD), and **(D)** superoxide dismutase (SOD) antioxidant activity of ‘Kinnow’ mandarin fruit during storage for 40 days at 5 ± 1°C and additional 5 days at 20 ± 5°C. Error bars denote the standard error (SE) of the mean. Means ± standard errors presented. To determine the interaction effects, factorial ANOVA was performed for the main factors, treatment, and storage time. GA + ZnO-NP, gum arabic + zinc oxide nanoparticles; DW, dried weight; KMFR, ‘Kinnow’ mandarin fruit rind.

During the postharvest storage of horticultural crops, ROS cause severe damage to the cell membranes of fresh produce; thus, antioxidant defense enzymes have been reported to remove factors leading to degradation of fruit cell membrane (Xing et al., 2016, 2017). Hong et al. (2012) reported that lipid peroxidation was harmful to pulp cells; therefore, the application of ECs can be effective in controlling the accumulation of unsaturated fatty acids. Naturally, during oxidative stress, fruits protect themselves from damage caused by ROS by producing enzymatic antioxidant defense systems such as catalase (CAT), PAL, peroxidase (POD) and SOD antioxidant activity as well as a wide array of non-enzymatic antioxidants (Blokhina et al., 2003; Yoksan and Chirachanchai, 2010). Generally, the catalase antioxidant activity declined as the storage period increased (Figure 9A). It was observed that the catalase antioxidant activity of the coated fruit was higher than those of the control fruit during the storage period. Catalase activity was significantly (*p* < 0.0001) influenced by an interaction between the treatments and storage period (Figure 9A). At the end of the storage period, it was observed that control fruit exhibited the lowest catalase activity (9.33 U mL^–1^ min^–1^ DW), followed by gum arabic coating alone (13.73 U mL^–1^ min^–1^ DW), GA + ZnO-NP 0.25% (15.19 U mL^–1^ min^–1^ DW), GA + ZnO-NP 0.5% (16.15 U mL^–1^ min^–1^ DW), GA + ZnO-NP 1% (18.17 U mL^–1^ min^–1^ DW). Catalase has hyperoxidase activity, which catalyzes the dismutation of hydrogen peroxide into water and oxygen and prevents the accumulation of toxic levels of hydrogen peroxide (H_2_O_2_) formed as a by-product of metabolic processes (Amoako et al., 2015). Therefore, coating the ‘Kinnow’ mandarin fruit with gum arabic coating enhanced its ability to prevent excessive hydrogen peroxide formation. Notably, enriching the gum arabic coating with ZnO-NPs further enhanced the properties of the coating in maintaining high catalase antioxidant activity against ROS. According to Ali et al. (2021), ECs such as gum arabic conserve lower activity of antioxidant enzymes and prevent fruit tissues from active oxygen, thus, leading to an increased storage period of coated fruits. Therefore, it can be hypothesized that gum arabic enriched with ZnO-NPs modulated exposure of the ‘Kinnow’ mandarin fruit to oxygen, thereby decreasing accumulation of H_2_O_2_ and eventually delaying senescence of the fruit.

Phenylalanine ammonia-lyase belongs to the plant aromatic amino acid ammonia-lyase enzymes family and is a key enzyme in regulating carbon flow from primary to secondary metabolism (He et al., 2020). The PAL captures oxygen radical species through the synthesis of phenolic compounds, and an increase in PAL activity may be a biochemical marker for the resistance of fruits to environmental stress (Medda et al., 2020). In the postharvest of horticultural commodities, the PAL activity prepares defense against pathogens and physiological disorders (He et al., 2020; Medda et al., 2020). In this study, it was observed that the PAL antioxidant activity increased and thereafter, declined as the storage period was extended (Figure 9B). The interaction between the treatments and storage significantly (*p* < 0.0001) affected the PAL antioxidant activity. After 40 days of storage, it was observed that control fruit had the lowest PAL activity (7.83 U mL^–1^ min^–1^ DW), followed by gum arabic (12.7 U mL^–1^ min^–1^ DW), GA + ZnO-NP 0.25% (14.71 U mL^–1^ min^–1^ DW), GA + ZnO-NP 0.5% (15.66 U mL^–1^ min^–1^ DW), and the highest PAL antioxidant activity (17.26 U mL^–1^ min^–1^ DW) was observed in ‘Kinnow’ mandarin fruit coated with GA + ZnO-NP 1%. According to Aghdam et al. (2012), the activity of PAL and total phenol content are associated with chilling tolerance of fruits; thus, the high PAL antioxidant activity in coated fruit. Similar results were reported by Chen et al. (2018), who reported that *Ficus hirta* fruit coated with chitosan EC on ‘Xinyu’ tangerines during cold storage at 5°C and 90 ± 5% RH resulted in higher PAL antioxidant activity compared to the control. Therefore, from our results, it was observed that enriching gum arabic EC with ZnO-NPs resulted in higher PAL antioxidant activity during the 40 days storage of ‘Kinnow’ mandarin fruit.

Peroxidase (POD) oxidizes several compounds by using hydrogen peroxide. Several utilities of peroxidase include degradation of hydrogen peroxide, removal of toxic compounds, defense against insect herbivores and many other stress-related responses in plants and fruits. It is also known to regain its activity during the storage of stored foods (He et al., 2020). Peroxidase is also involved in deteriorative changes in flavor, texture and color in raw and processed fruits and vegetables (Singh et al., 2015). In this study, the peroxidase activity increased initially, followed by a decline during the storage period (Figure 9C). The significant interaction (*p* < 0.0001) between the treatments and the storage period affected the peroxidase antioxidant activity of the stored ‘Kinnow’ mandarin fruit. The coating treatments enhanced peroxidase activity in ‘Kinnow’ mandarin compared to the control fruit. Therefore, ‘Kinnow’ mandarin fruit coated with GA + ZnO-NP 1% had higher peroxidase enzyme activity (20.73 U mL^–1^ min^–1^ DW), followed by GA + ZnO-NP 0.5% (18.34 U mL^–1^ min^–1^ DW), GA + ZnO-NP 0.25% (16.91 U mL^–1^ min^–1^ DW), gum arabic coating alone (15.86 U mL^–1^ min^–1^ DW) and the lowest peroxidase activity (9.9 U mL^–1^ min^–1^ DW) was observed in the control fruit (Figure 9C). Notably, incorporating the ZnO-NPs into the gum arabic matrix significantly (p < 0.05) improved the peroxidase activity compared to coating ‘Kinnow’ mandarin fruit with gum arabic coating alone. Ali et al. (2021) reported similar results that applying salicylic acid on stored ‘Kinnow’ mandarin fruit resulted in higher peroxidase antioxidant activity. The observed high peroxidase antioxidant activity also helps scavenge free radicals, which can damage cells under stress (Sels et al., 2008). The decline in enzyme activities after a specific period could be a consequence of its sensitivity to light and low-temperature liability (Masia, 1998).

Superoxide dismutase (SOD) catalyzes the dismutation of superoxide radicals to hydrogen peroxide (H_2_O_2_) and molecular oxygen, while catalase and ascorbate peroxidase (APX) are involved in eliminating H_2_O_2_ (Blokhina et al., 2003; Stephenie et al., 2020). In this study, the SOD antioxidant activity was significantly (*p* < 0.0001) influenced by the interaction between the treatments applied and the storage duration (Figure 9D). It was observed that coating the ‘Kinnow’ mandarin fruit significantly (*p* < 0.05) resulted in higher SOD antioxidant activity than the control. Enriching the gum arabic EC with ZnO-NPs improved SOD antioxidant activity during the 40 days storage of ‘Kinnow’ mandarin fruit. Higher SOD activity was maintained with increased ZnO-NPs in the gum arabic matrix. Hence, the highest SOD antioxidant activity (14.49 U mL^–1^ min^–1^ DW) was observed in ‘Kinnow’ mandarin fruit coated GA + ZnO-NP 1%, followed by GA + ZnO-NP 0.5% (12.98 U mL^–1^ min^–1^ DW), GA + ZnO-NP 0.25% (11.31 U mL^–1^ min^–1^ DW), gum arabic coating alone (9.19 U mL^–1^ min^–1^ DW) and lowest SOD antioxidant activity (7.66 U mL^–1^ min^–1^ DW) was observed in control fruit. Chen et al. (2018) reported similar findings on ‘Nanfeng’ mandarins coated with alginate-based EC containing *Ficus hirta* Vahl. fruit extract. According to the authors, increased antioxidant and defence-related enzymes such as SOD were reported. Thus, our results may imply that these antioxidant and defence-related enzymes were collectively induced by ZnO-NPs in gum arabic matrix to enhance antioxidant enzyme activity and prolong the shelf life of ‘Kinnow’ mandarin fruit. The observed results may also be linked to the known activity of zinc oxide as an antimicrobial and antioxidant agent. The mechanism of action of ZnO-NPs includes induction of oxidative stress, membrane disorganization of bacterial cell walls and release of zinc ions that bind to the membrane of microorganisms (Sawai et al., 1998; Sawai, 2003; Adams et al., 2006), thus preventing the accumulation of ROS in the presence of moisture. It can be hypothesized that incorporating ZnO-NPs in gum arabic matrix could potentially promote the activity of antioxidant enzymes and extend the shelf life of the coated fruit.

### Physiological Disorders

#### Rind Pitting Incidence and Severity

**FIGURE 10:**
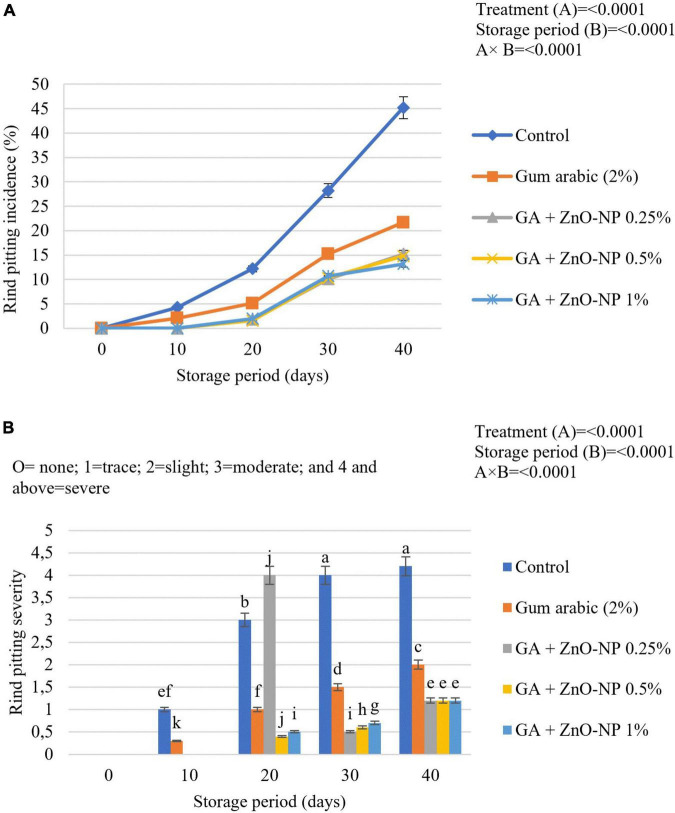
**(A)** Rind pitting incidence and **(B)** rind pitting severity of ‘Kinnow’ mandarin fruit during storage for 40 days at 5 ± 1°C and additional 5 days at 20 ± 5°C. Error bars denote the standard error (SE) of the mean. Means ± standard errors presented. To determine the interaction effects, factorial ANOVA was performed for the main factors, treatment, and storage time. GA + ZnO-NP, gum arabic + zinc oxide nanoparticles. Bars followed by different letters are significantly different at *p* < 0.05 according to Duncan’s multiple range tests.

Rind pitting physiological disorder is characterized by the emergence of clusters of collapsed oil glands scattered over the fruit’s surface. In due course, the affected region becomes bronze and develops more predominantly near the blossom end of the fruit (Agustí et al., 2001; Mothapo et al., 2018). Citrus fruit marketability depends on external appearance and rind quality (Magwaza et al., 2014); thus, it is important to maintain rind quality during the postharvest handling phase. There was a significant interaction (*p* < 0.0001) between the treatment and storage period for rind pitting incidence (Figure 10A). After 10 days in cold storage, 4.3% rind pitting incidence was observed in control (uncoated) fruit, while gum arabic coated fruit had 2.1% incidence. Whereas, fruit coated with gum arabic coating enriched with ZnO-NPs developed rind pitting incidence after 20 days of storage. After 40 days of storage, the highest rind pitting incidence (45.2%) was observed in control fruit, followed by gum arabic coating alone (21.7%), GA + ZnO-NP 0.25% (15.2%), GA + ZnO-NP 0.5% (14.8%), and GA + ZnO-NP 1% (13.2%). The rind pitting severity was also significantly (*p* < 0.0001) affected by the interaction between the treatments and the storage period. No significant (*p* > 0.05) differences were observed in ‘Kinnow’ mandarin fruit coated with gum arabic coating enriched with ZnO-NPs at the end of the storage period. Rind pitting severity was observed after 10 days of storage on both the control (1) and the fruit treated with gum arabic coating alone (0.3) (Figure 10B). After 40 days of storage, the highest rind pitting severity (4.2) was observed in the control fruit, followed by gum arabic coating alone (2.5), with slightly above trace rind pitting severity for GA + ZnO-NP 0.25% (1.2), GA + ZnO-NP 0.5% (1.2), and GA + ZnO-NP 1% (1.2). Similar results were reported by Shinga et al. (2021), who reported that carboxymethyl cellulose (CMC) encapsulated with moringa leaf extract (MLE) (CMC 1% + MLE 10%) controlled rind pitting incidence and severity in ‘Marsh’ grapefruit (*Citrus × paradisi*) better than the control. The reduction in rind pitting incidence and severity might be attributed to stimulated antioxidant enzymes, which results in enhanced antioxidant capacity of the fruit during storage, and in turn, reduced ROS production, and consequently alleviated the development of physiological disorders such as rind pitting (Cronjeì et al., 2017). Therefore, enriching the gum arabic coating with ZnO-NPs improved the defense ability of the mandarin fruit against oxidative stress during the storage period.

#### Chilling Injury Incidence and Severity

**FIGURE 11:**
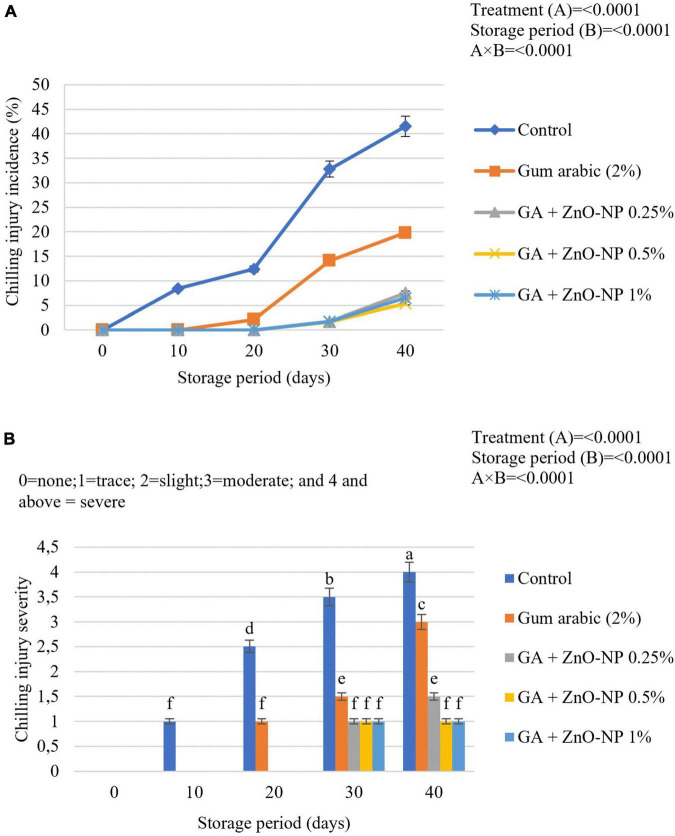
**(A)** Chilling injury incidence and **(B)** chilling injury severity of ‘Kinnow’ mandarin fruit during storage for 40 days at 5 ± 1°C and additional 5 days at 20 ± 5°C. Error bars denote the standard error (SE) of the mean. Means ± standard errors presented. To determine the interaction effects, factorial ANOVA was performed for the main factors, treatment, and storage time. GA + ZnO-NP, gum arabic + zinc oxide nanoparticles. Bars followed by different letters are significantly different at *p* < 0.05 according to Duncan’s multiple range tests.

Chilling injury is a physiological disorder that affects chill-sensitive horticultural crops stored at low temperatures. Sunken lesions on the rind of citrus fruits develop and slowly expand with time (Schirra et al., 1998). Fruit at cold storage did not manifest chilling injury symptoms; however, it became evident during simulated marketing conditions. Chilling injury severity and incidence increased with progressive storage of ‘Kinnow’ mandarin in all the treatments. The chilling injury incidence was significantly (*p* < 0.0001) influenced by the interaction of both the treatments and the storage period (Figure 11A). The chilling injury incidence (8.4%) was visible in the control fruit after 10 days of storage. ‘Kinnow’ mandarin coated with gum arabic EC alone had chilling injury incidence symptoms (2.1%) after 20 days of storage. ‘Kinnow’ mandarin coated with gum arabic enriched with ZnO-NPs had chilling injury incidence symptoms after 30 days of storage. At the end of the storage period, the highest chilling injury incidence (41.5%) was observed in control fruit, followed by gum arabic coating alone (19.8%), GA + ZnO-NP 0.25% (7.5%), GA + ZnO-NP 1% (6.5%) and the lowest chilling injury incidence (5.4%) were observed in ‘Kinnow’ mandarin fruit coated with GA + ZnO-NP 0.5%. This could be attributed to the ability of the composite coating to enhance cold acclimation of the stored ‘Kinnow’ mandarin fruit. It was also important to assess the severity of chilling injury for ‘Kinnow’ mandarin fruit marketability. Chilling injury severity increased as the chilling injury incidence increased during the storage period (Figure 11B). The degree of chilling injury severity was significantly (*p* < 0.0001) influenced by the interaction between the treatments and the storage period. Concerning the treatments applied, the chilling injury severity was low in fruit treated with GA + ZnO-NP 05% and GA + ZnO-NP 1% throughout the storage period. Uncoated fruit reached a ‘severe’ state (41.5%) by the end of the storage, suggesting that the fruit could be deemed unmarketable. ‘Kinnow’ mandarin fruit coated with gum arabic coating alone reached a ‘moderate’ state (19.8%), while fruit coated with gum arabic enriched with ZnO-NPs only had ‘traces’ (7.5, 6.5, and 5.4% for GA + ZnO-NP 0.25%, GA + ZnO-NP 1% and GA + ZnO-NP 05%, respectively) of chilling injury. Cell membrane lipids of fruit subjected to cold storage undergo changes in physical state from liquid-crystalline to solid-gel state, leading to an increase in membrane permeability and ion leakage (Galindo et al., 2004). Application of ECs induces cold acclimation, which leads to maintenance of membrane fluidity at low temperature and reduces electrolyte leakage and skin browning (Barman et al., 2011; Riva et al., 2020), thereby decreasing the chilling injury symptoms.

### Correlation Matrix and Principal Component Analysis

Pearson correlation was used to investigate the relationship between selected attributes associated ‘Kinnow’ mandarin fruit maturity at 40 days of storage (Table 3). Significant (*p* < 0.05) strong correlations were revealed. These include significantly (*p* < 0.05) strong and positive correlations that BrimA index showed with TSS (*r*^2^ = 0.949). This relationship clearly showed that the BrimA index was influenced by an increase in TSS. This could be attributed to the conversion of sucrose to sugars, thus having free soluble sugar accumulation. Weight loss correlated positively with electrolyte leakage (*r*^2^=0.965) and negatively with fruit texture (*r*^2^=–0.967). This supports our previous discussion that an increase in electrolyte leakage reduces membrane integrity and eventual weight loss. It is worth noting that the ascorbic acid content correlated negatively with chilling injury (*r*^2^=–0.964). This concurs with our findings that a decrease in ascorbic acid content could result from the occurrence of physiological disorders such as chilling injury. Furthermore, chilling injury negatively and strongly correlated with POD (*r*^2^=–0.912), PAL (*r*^2^=–0.942), SOD (*r*^2^=–0.954), and CAT (*r*^2^=–0.931), suggesting that the activity of these antioxidant enzymes was reduced as a result of chilling injury, especially in uncoated fruit. Against rind pitting incidence, a positive, strong correlation was observed with respiration rate (*r*^2^ = 0.961) and chilling injury (*r*^2^ = 0.959), supporting our previous discussion that an increase in respiration and chilling injury can promote rind pitting incidence during long term storage. The correlation results matrix suggests that coating the ‘Kinnow’ mandarin fruit with gum arabic enriched with ZnO-NPs influenced the physicochemical and phytochemical properties, antioxidant capacity, and enzyme of the fruit during the 40 days of storage. As supported by literature and the obtained results, the rate of respiration and moisture loss is a critical postharvest component that determines citrus fruits’ susceptibility to physiological disorders such as chilling injury and rind pitting incidence. These findings corroborate Shinga et al. (2021), who reported that mass loss significantly correlated positively with ‘Marsh’ grapefruit physiological disorders, and high CCI had a strong positive correlation with rind pitting incidence compared with low CCI. Ncama et al. (2016) also observed similar results after storing ‘Marsh’ grapefruit at 5°C for 6 weeks.

**TABLE 3:** Pearson’s correlation coefficients (*r*) matrix between quality attributes of uncoated and coated ‘Kinnow’ mandarin fruit stored for 40 days at 5 ± 1°C.

	BrimA	WL	REL	PI	RR	CCI	TSS	TA	FT	TFC	TPC	ABTS^+^	FRAP	AA	PW	JW	P:P	CI	POD	PAL	SOD
WL	–0.839	**1**																			
REL	**–0.945**	**0.965**	**1**																		
PI	**–0.905**	**0.981**	**0.988**	**1**																	
RR	**–0.969**	**0.908**	**0.964**	**0.961**	**1**																
CCI	**0.885**	**–0.963**	**–0.983**	**–0.968**	**–0.898**	**1**															
TSS	**0.949**	**–0.950**	**–0.976**	**–0.976**	**-0.989**	**0.924**	**1**														
TA	–0.708	0.857	0.794	0.778	0.738	–0.780	–0.825	**1**													
FT	**0.905**	**–0.967**	**–0.962**	**–0.976**	**–0.970**	**0.914**	**0.993**	–0.850	**1**												
TFC	**0.947**	**–0.927**	**–0.973**	**–0.980**	**–0.992**	**0.923**	**0.980**	–0.704	**0.966**	**1**											
TPC	**0.966**	**–0.875**	**–0.953**	**–0.950**	**–0.990**	**0.892**	**0.962**	–0.643	**0.934**	**0.992**	**1**										
ABTS^+^	**0.872**	**–0.987**	**–0.971**	**–0.995**	**–0.949**	**0.947**	**0.972**	–0.797	**0.983**	**0.969**	**0.929**	**1**									
FRAP	**0.854**	**–0.998**	**–0.971**	**–0.989**	**–0.925**	**0.961**	**0.961**	–0.838	**0.976**	**0.945**	**0.897**	**0.994**	**1**								
AA	**0.930**	**–0.950**	**–0.973**	**–0.987**	**–0.989**	**0.923**	**0.990**	–0.757	**0.986**	**0.995**	**0.976**	**0.984**	**0.965**	**1**							
PW	**0.941**	**–0.972**	**–0.991**	**–0.987**	**–0.976**	**0.957**	**0.995**	–0.841	**0.989**	**0.975**	**0.950**	**0.980**	**0.978**	**0.984**	**1**						
JW	**0.863**	**–0.999**	**–0.974**	**–0.984**	**–0.922**	**0.967**	**0.962**	**–0.867**	**0.975**	**0.936**	**0.889**	**0.987**	**0.998**	**0.958**	**0.981**	**1**					
P:P	**0.968**	**–0.914**	**–0.960**	**–0.949**	**–0.990**	**0.894**	**0.994**	–0.817	**0.978**	**0.969**	**0.961**	**0.941**	**0.926**	**0.975**	**0.982**	**0.932**	**1**				
CI	**–0.986**	**0.915**	**0.986**	**0.959**	**0.980**	**–0.946**	**–0.978**	0.768	**–0.948**	**–0.973**	**–0.972**	**–0.934**	**–0.925**	**–0.964**	**–0.980**	**–0.932**	**–0.979**	**1**			
POD	**0.853**	**–0.967**	**–0.945**	**–0.982**	**–0.949**	**0.906**	**0.966**	–0.768	**0.982**	**0.967**	**0.929**	**0.994**	**0.979**	**0.985**	**0.964**	**0.966**	**0.935**	**–0.912**	**1**		
PAL	**0.887**	**–0.978**	**–0.972**	**–0.995**	**–0.962**	**0.940**	**0.978**	–0.780	**0.985**	**0.979**	**0.945**	**0.999**	**0.988**	**0.992**	**0.982**	**0.979**	**0.950**	**–0.942**	**0.995**	**1**	
SOD	**0.954**	**–0.854**	**–0.928**	**–0.932**	**–0.989**	0.851	**0.958**	–0.634	**0.933**	**0.985**	**0.995**	**0.918**	**0.879**	**0.973**	**0.937**	**0.869**	**0.961**	**–0.954**	**0.928**	**0.937**	**1**
CAT	**0.879**	**–0.962**	**–0.957**	**–0.988**	**–0.964**	**0.917**	**0.972**	–0.746	**0.981**	**0.982**	**0.951**	**0.993**	**0.976**	**0.993**	**0.970**	**0.963**	**0.945**	**–0.931**	**0.998**	**0.997**	**0.947**

*Values in bold are different from 0 with a significant level of alpha = 0.05. WL, weight loss; REL, rind electrolyte leakage; PI, pitting incidence; RR, respiration rate; CCI, citrus color index; TSS, total soluble solids; TA, titratable acidity; FT, fruit texture; TF, total flavonoid; TP, total phenolic; FRAP, ferric ion reducing antioxidant power; AA, ascorbic acid; PW, rind weight; JW, juice weight; P:P, rind to pulp; CI, chilling injury; POD, peroxidase; PAL, phenylalanine ammonia-lyase; SOD, superoxide dismutase; CAT, catalase.*

**FIGURE 12:**
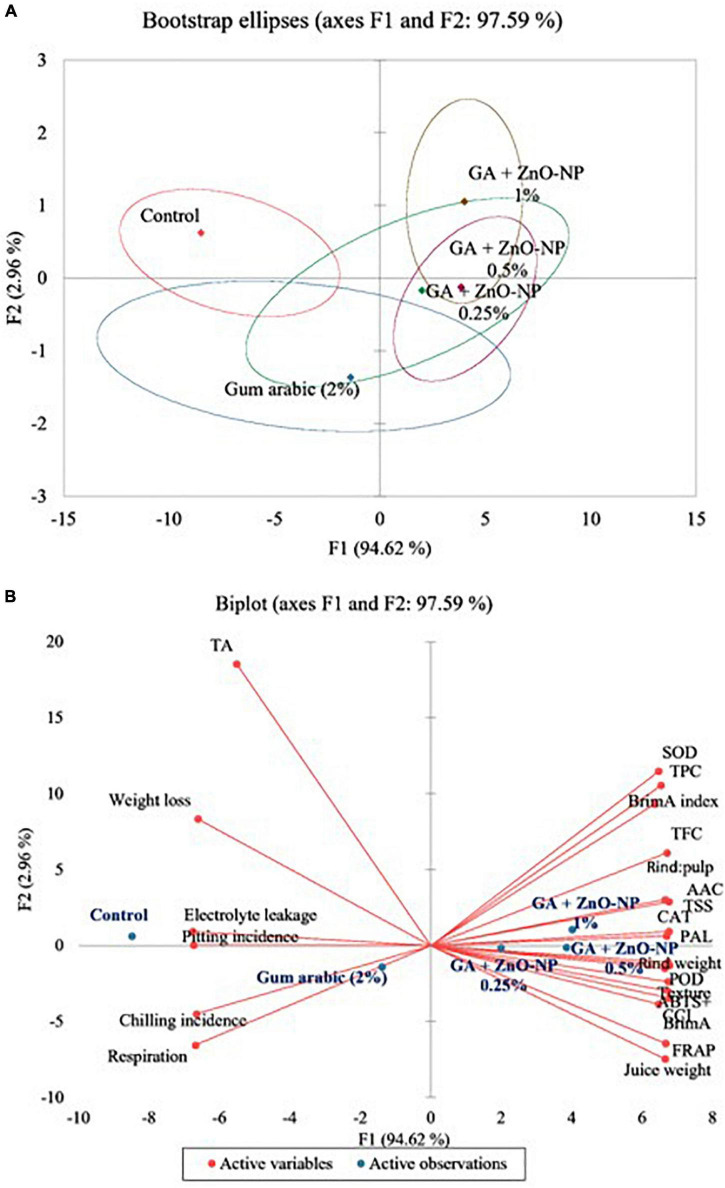
**(A)** Bootstrap ellipses of variance explained by each factor of the principal component and **(B)** principal component analysis showing active variables and observations for ‘Kinnow’ mandarin fruit. Data obtained at day 40 was used to generate the PCA.

To better understand the coating treatments and the evaluated parameters, a PCA bootstrap ellipses and biplot analysis were generated for data at 40 days of storage (Figure 12). The two factors (F1 and F2) of the PCA showed a high correlation of 97.59%, with F1 contributing 94.62% and F2 contributing 2.96%. Five detectable groups were evident from the bootstrap ellipses (Figure 12A). These included control, gum arabic (2%), GA + ZnO-NP 0.25%, GA + ZnO-NP 0.5%, and GA + ZnO-NP 1%. The PCA biplot further revealed the influence of each group against the parameters evaluated (Figure 12B). Positive scores of F1 corresponded with coating ‘Kinnow’ mandarin fruit GA + ZnO-NP 0.25%, GA + ZnO-NP 0.5%, and GA + ZnO-NP 1%. Negative scores along F1 corresponded with control and gum arabic (2%) treatments of ‘Kinnow’ mandarin fruit. Group 1 (control) was characterized by weight loss, rind pitting incidence and electrolyte leakage. Group 2 (gum arabic 2%) was closely linked to group 1 and was characterized by chilling injury incidence and respiration rate. Group 3 (GA + ZnO-NP 0.25%) and group 4 (GA + ZnO-NP 0.5%) were closely linked to group 5 (GA + ZnO-NP 1%), which was characterized by SOD, TPC, BrimA index, TFC, AAC, rind: pulp, TSS, CAT, and PAL. This suggests the positive role of the investigated ZnO-NPs in gum arabic matrix in maintaining the quality attributes of ‘Kinnow’ mandarin fruit during storage.

## Conclusion

Kinnow mandarin fruit has high nutritional and economic value; therefore, eco-friendly control measures to minimize postharvest quality loss of this essential fruit along the supply chain are necessary. Generally, the results from this study indicate that green synthesized ZnO-NPs in gum arabic matrix have the potential to maintain the quality of ‘Kinnow’ mandarin fruit and reduce postharvest physiological disorders associated with the fruit. The outcomes of this study showed that gum arabic (2% w/v) coating enriched with ZnO-NPs (0.5 and 1%) are prospective natural and eco-friendly postharvest treatments to reduce the incidence and severity of ‘Kinnow’ mandarin fruit rind disorders. Horticultural crops, like ‘Kinnow’ mandarin fruit, are marketed on a weight basis and visual appearance; thus, the investigated gum arabic enriched ZnO-NPs showed promise for extending the storage period of ‘Kinnow’ mandarin. In addition to alleviating rind disorders, the investigated coatings minimized metabolic rate, electrolyte leakage, and loss of fruit texture, resulting in overall fruit quality maintenance. This showed the uniqueness in the response of ‘Kinnow’ mandarin compared to other horticultural crops to the application of ZnO-NPs in gum arabic matrix. Based on the correlation matrix and PCA, it is recommended that gum arabic enriched with ZnO-NPs in the range of 0.5 and 1% be further optimized for commercial adoption as safe preservation technology to maintain the quality of ‘Kinnow’ mandarin fruit during storage.

## Data Availability Statement

The original contributions presented in this study are included in the article/supplementary material, further inquiries can be directed to the corresponding author.

## Author Contributions

OF: conceptualization, resources, project administration, and funding acquisition. KN: methodology, formal analysis, investigation, writing—original draft preparation, and writing—review and editing. KN and OF: software and validation. OF and OO: visualization, supervision, and writing—review and editing. All authors read and agreed to the published version of the manuscript.

## Author Disclaimer

The opinions, findings and conclusions, or recommendations expressed are those of the author(s) alone, and the funders accept no liability whatsoever in this regard.

## Conflict of Interest

The authors declare that the research was conducted in the absence of any commercial or financial relationships that could be construed as a potential conflict of interest.

## Publisher’s Note

All claims expressed in this article are solely those of the authors and do not necessarily represent those of their affiliated organizations, or those of the publisher, the editors and the reviewers. Any product that may be evaluated in this article, or claim that may be made by its manufacturer, is not guaranteed or endorsed by the publisher.
